# Persistent interferon signaling causes sensory neuron plasticity and pain before and during arthritis

**DOI:** 10.1038/s41593-026-02234-y

**Published:** 2026-03-10

**Authors:** Jie Su, Ming-Dong Zhang, Jussi Kupari, Dongoh Kwak, Laurence Picton, Bingze Xu, Leandro Flores do Nascimento, Yizhou Hu, Alejandro Gonzalez, Dmitry Usoskin, Zhongwei Xu, Marcin Szczot, Abdeljabbar El Manira, Rikard Holmdahl, Patrik Ernfors

**Affiliations:** 1 Department of Medical Biochemistry and Biophysics, Division of Molecular Neurobiology, Karolinska Institutet, Stockholm, Sweden; 2 Department of Neuroscience, Karolinska Institutet, Stockholm, Sweden; 3 Department of Medical Biochemistry and Biophysics, Division of Immunology, Karolinska Institutet, Stockholm, Sweden; 4 Department of Biomedical and Clinical Sciences, Center for Social and Affective Neuroscience, Linköping University, Linköping, Sweden

**Keywords:** Pain, Neuroimmunology, Autoimmune diseases

## Abstract

Although inflammatory processes in rheumatoid arthritis have been described, mechanisms driving pain are poorly defined. Here, we used a multitude of approaches to uncover the neural basis and causes of inflammatory pain. We show in mice with cartilage autoantibody-induced arthritis that early immune activation and a cytokine storm were mainly driven by vascular cells and monocytes/macrophages in the dorsal root ganglion. However, persistently elevated interferons and receptor activation of the MNK1/MNK2–eIF4E signaling pathway at all disease phases caused sensory–motor dysfunction and pain by inducing hyperexcitability and sensitization of a GFRA3^+^ C-fiber subtype of joint-innervating sensory neurons. Signaling pathway inhibition in vivo reversed pain and restored limb function. Like mice, human sensory neurons expressed interferon receptors, and type 1 interferons and signaling were increased only in individuals with painful rheumatoid arthritis. The finding that joint pain before and during arthritis is caused by a defined cytokine and signaling pathway holds promise for targeted therapies for pain relief in arthritis.

## Abstract (web summary)

Su et al. show that inflammatory arthritis pain stems from sustained interferon-driven MNK1/MNK2–elF4E signaling that sensitizes joint-innervating neurons. Blocking this pathway reverses pain, suggesting a targeted therapeutic strategy.

## Main

Emerging data show that in many inflammatory conditions, pain develops before onset of inflammation and persists after inflammation resolves, indicating that it is not merely an accompanying symptom of inflammation. Joint pain (arthralgia) typically appears before the onset of rheumatoid arthritis (RA) and persists irrespective of arthritis severity. Effective treatment of arthritis often fails to cure pain; ‘remaining pain’ affects up to 25% of individuals and causes substantial suffering^1^.

Pain associated with inflammation has been ascribed to increased excitability of sensory neurons in the dorsal root ganglia (DRGs)^2^. Activity in primary sensory neurons is essential because local anesthesia relieves acute and chronic pain^3^. RA is associated with a strong autoantibody response and production of multiple cytokines, including tumor necrosis factor (TNF), interleukin-1β (IL-1β) and IL-6, which contribute to inflammatory disease. Cartilage-binding autoantibodies and inflammatory cytokines induce DRG neuron hyperexcitability and sensitization to painful stimuli when injected in experimental animals^4–6^. Proposed mediators include Toll-like receptor ligands, inflammatory lipids, neuropeptides and growth factors^7–9^. In mice, single-cell RNA sequencing (scRNA-seq) has identified many sensory neuron types that generate pain behavior, and the human evolutionary homologs are largely known^10–14^. Most express neuropeptides, consistent with peptidergic fibers in joint tissues^15,16^, but which sensory neuron types are functionally involved in arthritis and how they are molecularly perturbed remain unclear. Thus, the relative importance of different inflammatory mediators, the identity of the pain-causing sensory neuron population and the intracellular mechanisms driving hyperexcitability and pain in arthritis are not fully understood.

Type 1 interferons (IFNs) orchestrate antiviral defenses. Serum type 1 IFN levels increase early in RA, but type 1 IFNs have not been considered essential for arthritis development^2,17^. We reasoned that identifying the neuron type that causes pain and mapping intercellular communication between immune and neuronal cells would reveal cellular and molecular mechanisms responsible for sensory dysfunction in arthritis. Our work shows that an alternative type 1 IFN signaling pathway drives hyperexcitability in molecularly defined joint afferent sensory neurons, causing joint pain, impaired dexterity and reduced limb function.

## Results

### Autoantibody-induced arthritis sensitizes multiple sensory neuron types before, during and after inflammation

**Fig. 1:**
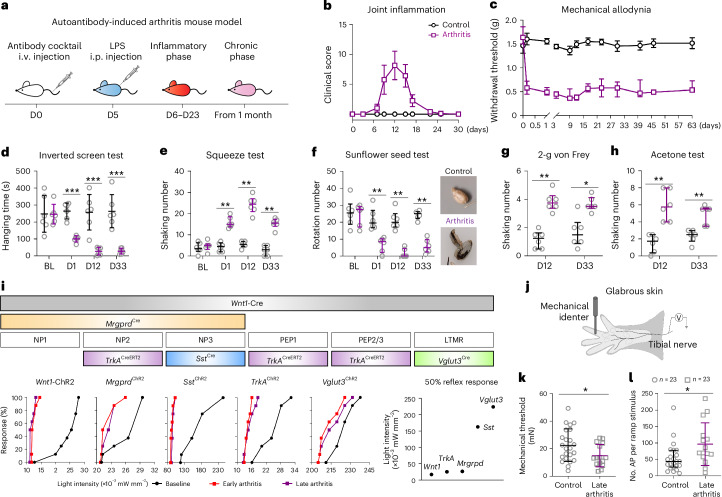
Characterization of pain behaviors in autoantibody-induced arthritis model. **a**, Cartilage autoantibody-induced arthritis mouse model. D, day; i.p., intraperitoneal; i.v., intravenous. **b**, Time course of transient joint inflammation (clinical score) from days 6 to 23 after autoantibody injection in C57BL/6N mice (*n* = 10 per group). LPS was injected on day 5 to enhance arthritis development. **c**, Time course of mechanical allodynia in mice with arthritis starting as early as 4 h and lasting until day 63 after autoantibody injection (*n* = 11 per group). **d**, Hanging time in the inverted screen test at different timepoints after antibody injection (D1, arthralgia; D12, arthritis with peak inflammation; D33, arthritis after inflammation remission) in control mice and mice with arthritis (*n* = 6 per group in **d***–***h**). BL, baseline control. **e**, Shaking numbers in the clip squeeze test after antibody injection. **f**, Rotation numbers in the sunflower seed test and seed images after peeling the shell to expose the seed kernel. **g**, Shaking numbers in the 2-g von Frey test. **h**, Shaking numbers in the acetone test. **i**, Top, Cre and Cre^ERT2^ mouse strains used in the study to target specific DRG neuron populations. Bottom, reflex response percentage to blue light stimulation in the different mouse strains (crossed with R26-ChR2 mice) at different stages after arthritis (early arthritis: early phase of autoantibody-induced arthritis during inflammation, days 9–21; late arthritis: late phase of arthritis after remission of inflammation, after day 30); light intensity for inducing reflex responses in 50% of mice from different strains (*n* = 7 or 8 per strain). *TrkA* is also known as *Ntrk1*; *Vglut3* is also known as *Slc17a8*. **j**, Skin nerve recording paradigm. **k**, Mechanical thresholds of mice injected with saline (‘control’) or autoantibody (around 3 months after antibody injection, ‘late arthritis’). **l**, Number of mechanically induced action potentials (AP) during the force ramp application. Individual recorded fibers were plotted for **k** and **l**. Data are expressed as mean ± s.e.m. (**b**,**c**), mean ± s.d. (**d**,**k**), or median with interquartile range (**e**–**h**,**l**). Differences between control and arthritis groups at different timepoints were analyzed by two-way analysis of variance (ANOVA) with Bonferroni’s multiple comparisons test (**d**), two-tailed unpaired *t*-test (**k**) or two-tailed nonparametric Mann–Whitney test (**e**–**h**,**l**). *P < 0.05, **P < 0.01 and ***P < 0.001. The exact and adjusted *P* values are listed in the Source data. Source data

**Extended Data Fig. 1:**
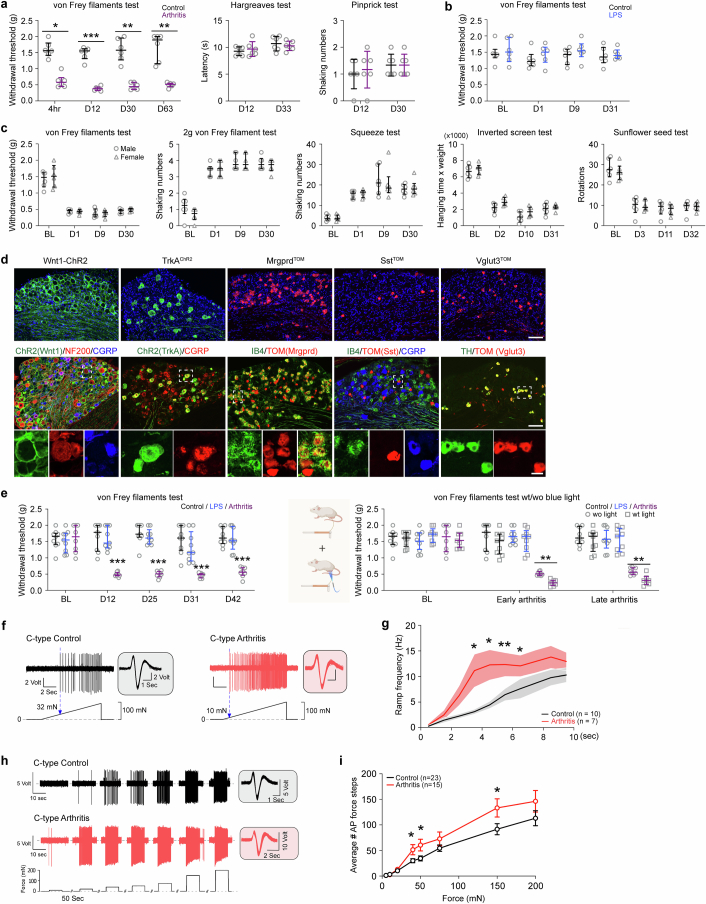
Pain behavior and skin-nerve recording in cartilage autoantibody-induced arthritis mouse. **a**, Mechanical allodynia, heat sensitivity and mechanical hyperalgesia in cartilage autoantibody-induced arthritis mice. **b**, Mechanical sensitivity in saline (Control) and LPS injected mice. **c**, Sex differences in arthritis-induced mechanical hypersensitivity, joint pain or motor-sensory function (C57BL/6 N, n = 6/group in **a**-**c**). **d**, Characterization of mouse strains Wnt1-ChR2, TrkA^ChR2^, Mrgprd^TOM^, Sst^TOM^ and Vglut3^TOM^ with immunohistochemistry comparing reporter expression (Tom) and markers for neuronal subtypes as indicated (n = 4 per strain). Scale bars = 100 μm and 20 μm for the inset. **e**, Left: Mechanical sensitivity in control, LPS alone and arthritis groups in TrkA^ChR2^ mice. Right: Effects of combined subthreshold blue light on mechanical threshold (n = 8/9 per group). Cartoon was created in BioRender. Fatt, M. (2026) https://BioRender.com/2wkmnd5. **f**, Representative C-type fiber recordings (CV < 1.2 m/s) from control and arthritis mice (‘C-type Arthritis’, 3 months after antibody injection; right panel). **g**, Mechanically induced firing frequencies of fibers from control and arthritis mice, thick lines represent mean number of action potentials in 1 second bins, shadowed regions are the s.e.m. **h**, Representative C-type fiber recordings from control and arthritis mice. **i**, Average number of mechanically induced action potentials. Data are expressed as mean with s.d. for Hargreaves and inverted screen tests, median with interquartile range for other behavioral tests or mean with s.e.m. for skin nerve recordings. Hargreaves test (**a**) and inverted screen test (**c**) data were analyzed with two-way ANOVA followed by Bonferroni’s multiple comparisons test, other behavioral tests data in (**a**-**c** and **e**) were analyzed with Kruskal-Wallis test followed by Dunn’s multiple comparisons test. von Frey filament tests wt/wo blue light data in (**e**) were analyzed with Wilcoxon matched-pairs signed rank test. Recording data were analyzed with two-tailed unpaired *t*-test. * indicates *P* < 0.05, ** indicates *P* < 0.01 and *** indicates *P* < 0.001. The exact and adjusted *P* values are listed in the source data. Source data

We used a cocktail of monoclonal pathogenic antibodies targeting cartilage, similar to RA autoantibodies, to induce arthritis in mice (cartilage autoantibody-induced arthritis model)^18^. Macroscopic arthritis (swelling and redness) developed between days 6 and 23 after injection of autoantibodies to native and citrullinated cartilage proteins, which was enhanced by lipopolysaccharide (LPS) on day 5 (Fig. 1a,b). Mice developed mechanical allodynia within 4 h, with reduced von Frey thresholds before inflammation (4 h, days 1 and 3), during inflammation (days 9, 12, 17 and 23) and long after resolution (days 30, 40, 46 and 63; Fig. 1c and Extended Data Fig. 1a). Thus, this model captures arthralgia before arthritis onset and after arthritis remaining pain.

To assess overall sensory–motor limb function, we used the inverted screen test^19^. Autoantibody-injected mice performed worse at all three disease phases (Fig. 1d). To model joint tenderness, we developed a paw squeeze test; autoantibody-injected mice showed increased pain-like behavior before, during and after inflammation (Fig. 1e). We quantified stiffness and loss of fine motor skills using the sunflower seed handling assay. Control mice grasped and rotated seeds efficiently, exposing intact kernels, whereas mice with arthritogenic autoantibodies at all phases failed to maintain grip and adopted inefficient alternative strategies, leading to partial peeling and failure to expose intact kernels (Fig. 1f and Supplementary Videos 1 and 2). These mice also showed increased nocifensive behavior to mechanical pricking (mechanical hyperalgesia) and cold hyperalgesia during and after inflammation (Fig. 1g,h). Sensitization occurred before LPS injection (4 h, day 1 and day 3), and LPS alone neither increased nociceptor excitability nor pain behavior, indicating that cartilage autoantibodies, not LPS or arthritis itself, drive pain behavior and sensory–motor dysfunction (Extended Data Fig. 1a,b). We observed similar allodynia, hyperalgesia, joint pain, dexterity loss and limb dysfunction in males and females (Extended Data Fig. 1c). We conclude that autoantibodies cause reduced limb function, arthralgia, impaired dexterous forepaw use and cutaneous mechanical and cold hyperalgesia before, during and after inflammation, paralleling clinical observations in RA^20,21^.

We next asked whether autoantibody-induced arthritis leads to sensory nerve hyperexcitability and which neuronal types are affected. We used the following Cre driver lines to target molecularly defined sensory neuron subtypes identified by scRNA-seq^12^: *Wnt1*-Cre (all sensory neurons^22^), *Mrgprd*^Cre^ (C-high-threshold mechanoreceptors (HTMRs)/pruriceptor subtypes NP1, NP2 and NP3 (ref. ^23^)), *TrkA*^CreERT2^ (C-high-threshold heat/mechanoreceptor subtype PEP1, A-HTMR subtype PEP2, A-HTMR subtype PEP3 and C-HTMR/pruriceptor subtype NP2 (ref. ^24^)), *Sst*^Cre^ (C-HTMR/pruriceptor subtype NP3 (ref. ^25^)) and *Vglut3*^Cre^ (C-low-threshold mechanoreceptors (C-LTMRs)^26^; Fig. 1i). Reporter crosses confirmed expected recombination patterns (Extended Data Fig. 1d). We then crossed each line to Ai32 to obtain *Wnt1*-channelrhodopsin2 (*Wnt1*-ChR2), *Mrgprd*^ChR2^, *TrkA*^ChR2^, *Sst*^ChR2^ and *Vglut3*^ChR2^ mice. We assessed excitability by applying increasing light intensities to the paw and measuring withdrawal thresholds.

In control mice, thresholds differed markedly across neuron types: *Wnt1*-ChR2, *TrkA*^ChR2^ and *Mrgprd*^ChR2^ mice had low thresholds (13 × 10^−3^–21 × 10^−^^3^ mW mm^−^^2^), whereas *Sst*^ChR2^ and *Vglut3*^ChR2^ mice required >100 × 10^−^^3^ mW mm^−2^. Autoantibody-induced arthritis decreased thresholds and increased excitability in all neuron types during and after arthritis, with *Wnt1*-ChR2 and *TrkA*^ChR2^ mice showing the largest shifts (Fig. 1i). LPS alone did not affect excitability (Extended Data Fig. 1e and Supplementary Fig. 1). Thus, autoantibody exposure induces broad sensory dysfunction across neuron subtypes, consistent with the multimodal sensory–motor phenotype.

TRKA^+^ populations include mechanosensitive C-fiber nociceptors. To directly measure action potential threshold and firing, we performed skin nerve recordings of mechanosensitive C-fibers (Fig. 1j). In autoantibody-treated mice, C-fibers had lower mechanical thresholds and higher firing frequency and total action potentials during a 0- to 100-mN force ramp stimulus, with increased firing at relatively low forces (Fig. 1k,l and Extended Data Fig. 1f,g). Force step stimuli at 10–200 mN increased firing starting already at 40 mN in mice with autoantibodies (Extended Data Fig. 1h,i). We conclude that autoantibodies cause C-fiber axon hyperexcitability, lowering activation thresholds and enhancing suprathreshold responses.

### Molecularly defined joint-innervating neurons mediate arthritis pain

**Fig. 2:**
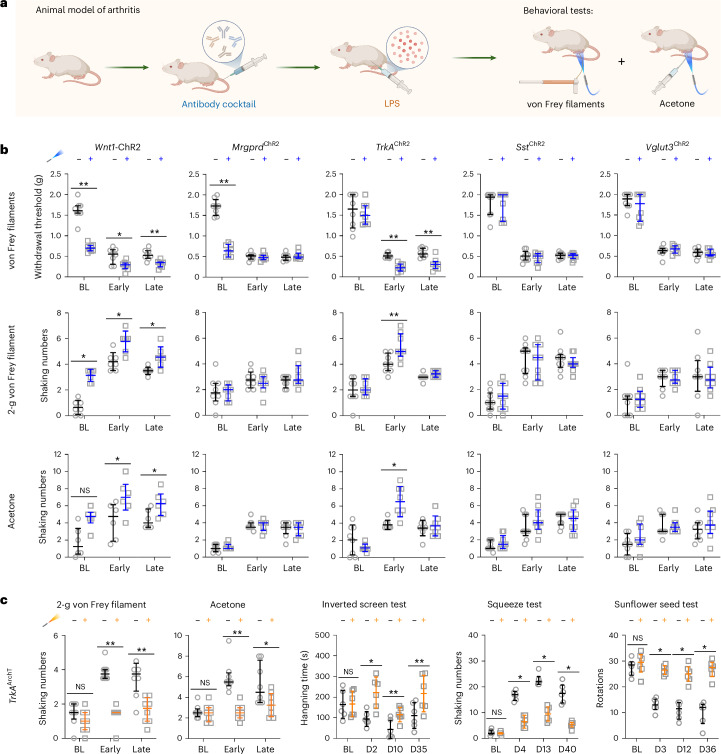
TRKA^+^ sensory neurons contribute to arthritis pain behavior. **a**, Paradigm for optogenetic pain-like behavioral analysis in mice with antibody-induced arthritis. Created in BioRender; Fatt, M. https://biorender.com/2wkmnd5 (2026). **b**, Subthreshold photostimulation with blue light combined with naturalistic stimuli (mechanical threshold by von Frey filaments, mechanical pricking by 2-g von Frey filament and acetone test) before inducing arthritis (BL) and early (during inflammation) and late arthritis (after inflammatory remission) in *Wnt1*-ChR2, *Mrgprd*^ChR2^, *TrkA*^ChR2^, *Sst*^ChR2^ and *Vglut3*^ChR2^ mice (*n* = 7 or 8 mice per strain). **c**, Mechanical/cold sensitivity and joint pain tests (including inverted screen, squeeze and sunflower seed tests) in *TrkA*^ArchT^ mice before and after yellow light (566 nm) application (0.1 mW mm^−2^, 30 min) at different timepoints after inducing arthritis (*n* = 8 for mechanical/cold sensitivity, *n* = 6 for joint pain tests). Data are expressed as mean ± s.d. for the inverted screen test in **c** or median with interquartile range for the rest of the assays. Differences in stimulation without and with LED light at different timepoints were analyzed by two-tailed paired *t*-test for the inverted screen test or Wilcoxon matched-pairs signed rank test for other assays. *P < 0.05 and **P < 0.01. NS, not significant. The exact *P* values are listed in the Source data. Source data

Although many sensory neuron types became hyperexcitable, we hypothesized that a specific subset drives the pain phenotypes. We combined subthreshold optogenetic activation with behavioral assays in *Wnt1*-ChR2, *Mrgprd*^ChR2^, *TrkA*^ChR2^, *Sst*^ChR2^ and *Vglut3*^ChR2^ mice. We adjusted light intensity for each line so that light alone did not evoke behavior and assessed von Frey thresholds and nocifensive responses to 2-g von Frey pricking and cold (acetone) during early (arthralgia with inflammation) and late (postinflammation) phases (Fig. 2a). Autoantibodies induced allodynia and hyperalgesia in all lines, but subthreshold light potentiated allodynia and nocifensive responses only in *TrkA*^ChR2^ and *Wnt1*-ChR2 mice (Fig. 2b).

**Extended Data Fig. 2:**
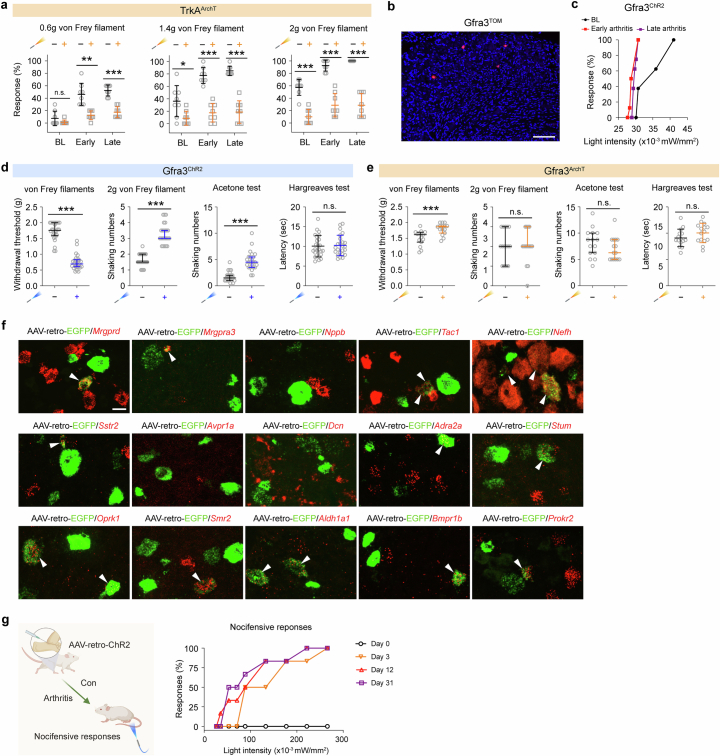
The role of GFRA3^+^ C-fiber nociceptors in normal mechanical and cold sensation, and characterization of retrograde tracing neurons from ankle joint. **a**, In control TrkA^ArchT^ mice, exposure to yellow light (0.1 mWatt/mm^2^, 30 min) caused less percentage of reflex reaction to 1.4 g and 2.0 g von Frey filaments indicating that TrkA neurons contribute to noxious, but not innocuous (0.6 g) pricking. Inhibition of TrkA^+^ neurons through stimulation of yellow light (0.1 mWatt/mm^2^, 30 min) completely reversed the increased sensitivity and reflex reactions in both early and late phases of arthritis (n = 8). **b**, Gfra3^+^ neurons (Tom) in DRGs of Gfra3^TOM^ mice, DAPI (blue) is nuclear staining. Scale bar = 100 μm. **c**, Percentage of reflex responses in mice to blue light at different light intensities in Gfra3^ChR2^ mice, with left-shifted response curve in early and late phases of arthritis (n = 8). **d**, In Gfra3^ChR2^ mice, a combination of blue light (subthreshold for reflex: 30.2×10^−3^ mWatt/mm^2^; subthreshold for nocifensive: 39.6×10^−3^ mWatt/mm^2^) increased mechanical and cold sensitivity with lower mechanical threshold and more nocifensive responses to 2 g von Frey filament and acetone test but had no effect on thermal sensitivity (latency in Hargreaves) (n = 24). **e**, In Gfra3^ArchT^ mice, inhibition by yellow light (0.1 mWatt/mm^2^, 45 min) led to higher mechanical withdrawal threshold in von Frey test compared to before exposure to yellow light (n = 16). **f**, Representative RNAscope images of traced joint innervating sensory neurons and marker genes for different DRG subpopulations. Scale Bar: 20 μm. Arrowheads indicate co-localization. **g**, Correlations between light intensity and percentage of nocifensive responses in Control and Arthritis mice (multiple timepoints) injected with AAV-retro-ChR2 virus. Cartoon was created in BioRender. Fatt, M. (2026) https://BioRender.com/2wkmnd5. Data are expressed as mean with s.d. in (a) and Hargreaves test in (d), or median with interquartile range for other behavioral tests. Wilcoxon matched-pairs signed rank test was applied for von Frey filaments, 2 g von Frey filament and acetone tests, whereas Hargreaves test was analyzed by paired *t* test (two-tailed) in (**a**, **d**, **e**). * indicates *P* < 0.05, ** indicates *P* < 0.01, *** indicates *P* < 0.001 and n.s. means no significance. The exact *P* values are listed in the source data. Source data

To establish the importance of TRKA^+^ sensory neurons, we crossed *TrkA*^CreERT2^ mice with Ai40 (ArchT) mice to silence TRKA^+^ neurons (*TrkA*^ArchT^). Optogenetic inhibition reversed allodynia and pricking and cold hyperalgesia, as well as joint pain behavior, limb dysfunction and paw dexterity deficits during and after inflammation (Fig. 2c and Extended Data Fig. 2a).

**Fig. 3:**
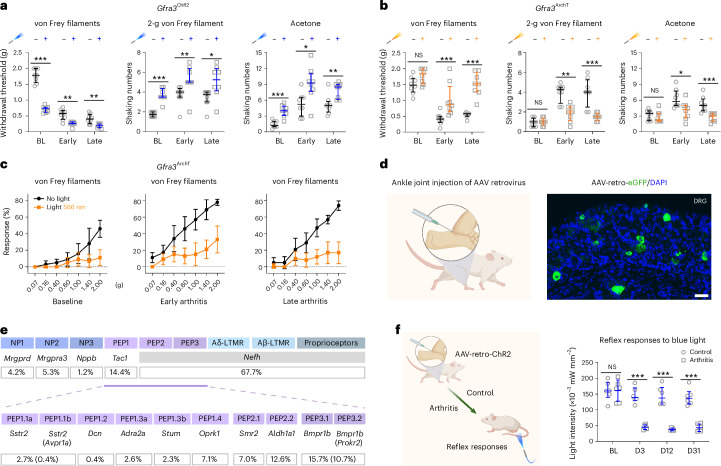
GFRA3^+^ C-nociceptors as a cause for pain behavior in arthritis. **a**, Mechanical withdrawal threshold (von Frey filaments test) and nocifensive responses (shaking numbers) to 2-g von Frey filament and acetone tests without light or with subthreshold blue light (470 nm) in *Gfra3*^ChR2^ mice (*n* = 8); early, inflammatory phase of arthritis; late, postinflammatory phase of arthritis. **b**, Mechanical and cold sensitivity in *Gfra3*^ArchT^ mice (*n* = 8) before and after yellow light (566 nm) application (0.1 mW mm^−2^, 45 min). **c**, Percentage of reflex response to different von Frey filaments without or with yellow light applied (0.1 mW mm^−2^, 45 min) before (baseline) and after antibody-induced arthritis (early and late phases of arthritis; *n* = 8). **d**, Retrograde tracing of sensory neurons innervating the ankle joint by intra-articular injection of AAV-retro-GFP virus (*n* = 4 mice). DAPI was used as a counterstain. Scale bar, 50 µm. **e**, Summary of identities of traced neurons with DRG neuronal subpopulation marker genes (*n* = 2 mice). **f**, Light threshold for withdrawal reflex responses in control mice and mice with arthritis injected with AAV-retro-ChR2 virus (*n* = 6 per group). Cartoons in **d** and **f** were created in BioRender; Fatt, M. https://biorender.com/2wkmnd5 (2026). Data are expressed as median with interquartile range (**a**,**b**) or mean ± s.d. (**c**,**f**). Data were analyzed with a two-tailed Mann–Whitney test (**a**,**b**) or two-way ANOVA with Bonferroni’s multiple comparisons test (**f**). *P < 0.05, **P < 0.01 and ***P < 0.001. The exact and adjusted *P* values are listed in the Source data. Source data

TRKA marks several sensory neuron types (PEP1, PEP2, PEP3 and NP2). We next asked whether PEP1 C-fiber neurons drive arthritis pain. GFRA3 is expressed exclusively in PEP1 neurons^12^. We generated *Gfra3*^CreERT2^ mice with a conditional reporter, ChR2 or ArchT alleles (*Gfra3*^TOM^, *Gfra3*^ChR2^ and *Gfra3*^ArchT^). *Gfra3*^TOM^ confirmed DRG recombination (Extended Data Fig. 2b). C-Mechanonociceptors in *Gfra3*^ChR2^ mice became hyperexcitable after autoantibody treatment, with reduced light-induced withdrawal thresholds during and after inflammation (Extended Data Fig. 2c). Subthreshold light markedly potentiated allodynia and pricking and cold pain behavior in *Gfra3*^ChR2^ mice (Fig. 3a). Silencing GFRA3^+^ neurons in *Gfra3*^ArchT^ mice reversed allodynia and pricking and cold hyperalgesia during and after inflammation (Fig. 3b). In control mice, silencing GFRA3^+^ neurons primarily affected behavior to noxious mechanical forces, whereas in arthritis, it reversed both allodynia (≤1.4 g) and noxious responses (>1.4 g; Fig. 3c). Thus, arthritis-sensitized GFRA3^+^ neurons contribute to both allodynia and heightened pain sensitivity.

Although the contribution of TRPV1- and CALCA (CGRP)-expressing sensory neurons to normal pain behavior has previously been studied^27^, they mark multiple sensory neuron types (PEP1 C-HTMRs, PEP2/PEP3 A-HTMRs and NP class C-HTMRs/pruriceptors), so PEP1 function in naive mice was unclear. In *Gfra3*^ChR2^ mice, subthreshold light combined with natural stimuli revealed GFRA3^+^ neuron contributions to mechanical threshold detection and nocifensive responses to mechanical and cold stimuli but not heat thresholds (Extended Data Fig. 2d). Silencing *Gfra3*^ArchT^ neurons in naive mice caused only a small shift in mechanical threshold (Extended Data Fig. 2e), suggesting that other neuron types compensate. We conclude that in naive mice, loss of GFRA3^+^ activity minimally affects baseline pain behaviors, but in mice with arthritogenic autoantibodies, silencing GFRA3^+^ neurons suffices to fully reverse allodynia and pain.

To define the molecular identity of joint-innervating subtypes of sensory neurons, we injected AAV-retro-CAG-eGFP intra-articularly into the ankle and labeled ~7.5% of L3–L6 DRG neurons (Fig. 3d). RNAscope with sensory neuron subtype markers^10^ combined with green fluorescent protein (GFP) showed that joint afferents include C- and A-fiber PEP neurons. Quantification revealed that PEP1.4^*Oprk1*^ C-fiber (7.1% of GFP^+^), PEP2.2^*Aldh1a1*^ A-fiber (12.6%) and PEP3.2^*Bmpr1b*/*Prokr2*^ A-fiber (10.7%) neurons dominate joint innervation, with smaller contributions (2–3%) from PEP1.1a^*Sstr2*^, PEP1.3a^*Adra2a*^ and PEP1.3b^*Stum*^ C-fiber neurons (Fig. 3e and Extended Data Fig. 2f).

To directly test whether joint afferents become hyperexcitable after autoantibody treatment, we expressed ChR2 in articular afferents by intra-articular injection of AAV-retro-hSyn1-ChR2 and recorded light–response relationships at baseline and days 3, 12 and 31 after saline or autoantibody treatment (Fig. 3f). Both groups showed reflex withdrawal at baseline, but autoantibody-treated mice showed a marked reduction in light intensity needed to trigger withdrawal at all later timepoints (Fig. 3f). Light activation of articular afferents did not evoke nocifensive behavior at any intensity in saline-treated mice, but autoantibody-treated mice displayed robust nocifensive responses at all postinjection timepoints (Extended Data Fig. 2g). Thus, in naive mice, joint afferent activation triggers protective reflexes, whereas in autoantibody-exposed mice, the same afferents produce pain-like behavior.

### Type 1 IFNs drive a transient multicellular inflammatory reaction in the DRG

**Extended Data Fig. 3:**
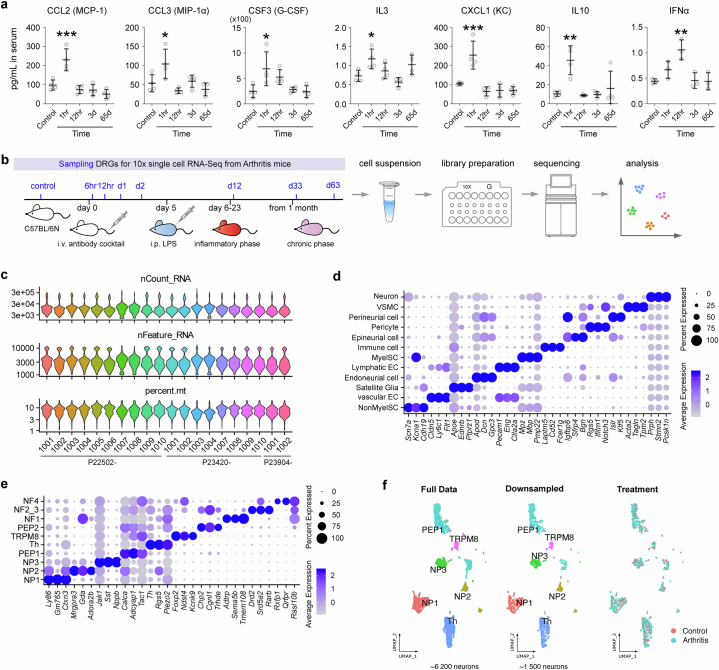
Single-cell RNA sequencing of DRG neurons in cartilage autoantibody-induced arthritis mice. **a**, Cytokine expression in sera collected at different times (hr, hours; d, days) after autoantibody injection and from control mice (n = 3 or 4 per group). **b**, Illustration of sampling and workflow for scRNA-seq of DRG cells from control and autoantibody injected mice. **c**, Sample metrics violin plots after initial quality control for scRNA-seq data. **d**, Dot plot of top 3 marker genes for each major cell type of sequenced cells in DRGs. **e**, Dot plot of top 3 marker genes for each DRG neuronal cell type. **f**, UMAP of DRG neuronal clusters and of randomly sampled (25%) dataset used for co-regulated gene module analysis showing intermingled distribution of cells from control and arthritis samples. Data are expressed as mean with s.d. in (**a**). Differences were analyzed by ordinary one-way ANOVA followed by Dunnett’s multiple comparisons test. * indicates *P* < 0.05, ** indicates *P* < 0.01 and *** indicates *P* < 0.001. The adjusted *P* values are listed in the source data. Source data

Individuals with RA show increased circulating cytokines^28^. Similarly, autoantibody-injected mice showed rapid (within 1 h) serum increases in IL-3, IL-10, IFNα, CSF3, CCL2, CCL3 and CXCL1, which largely normalized by 12 h (Extended Data Fig. 3a), implying that sustained sensitization beyond the acute phase depends on local tissue activity. The very rapid sensitization before overt joint inflammation (Fig. 1b,c and Extended Data Fig. 1a) supports pain independent of joint inflammation.

**Fig. 4:**
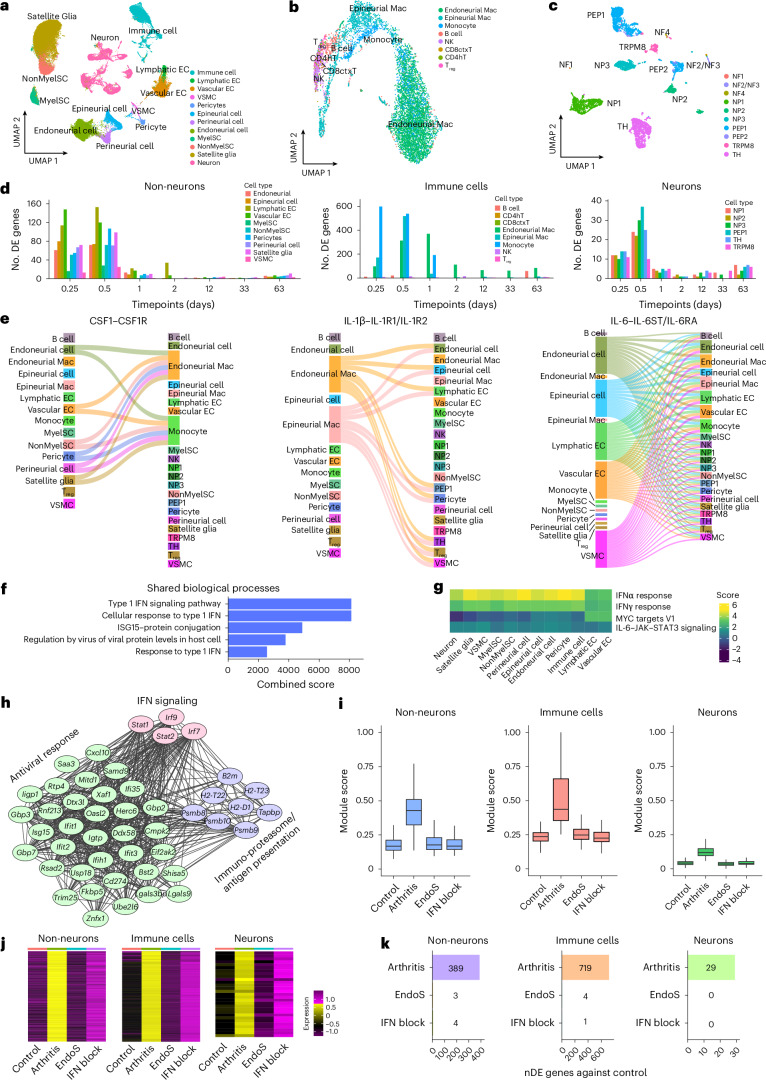
Acute cytokine storm in the DRG caused by type 1 IFNs. **a**, Uniform manifold approximation and projection (UMAP) showing the distribution of cell clusters (86,052 cells) from scRNA-seq of DRGs from control mice and mice with arthritis. EC, endothelial cells; MyelSC, myelinating Schwann cells; NonMyelSC, nonmyelinating Schwann cells; VSMC, vascular smooth muscle cells. **b**, UMAP showing the distribution of immune cells. CD4hT, CD4^+^ helper T cells; CD8ctxT, CD8^+^ cytotoxic T cells; Mac, macrophages; T_reg_, regulatory T cells. **c**, UMAP of neuronal clusters (6,200 cells) from scRNA-seq of DRGs from control mice and mice with arthritis. TH, tyrosine hydroxylase (a marker for C-LTMRs in the DRG). **d**, Number of differentially expressed (DE) genes in DRG cell types in mice with arthritis. **e**, Ligand–receptor interaction analysis of new interactions in the DRG 12 h after autoantibody injection. **f**, Combined score of pseudobulk Gene Ontology analyses at 12 h showing the relation to the type 1 IFN signaling pathway and response to IFNs. **g**, Top GSEA pathways at the 12-h data point. **h**, STRING network visualization of the co-regulated modules at 12 h in neurons. **i**, Module score of different types of cells in the DRG 12 h after antibody injection as well as endoglycosidase S (EndoS)-treated autoantibody-injected mice and mice with arthritis preceded by antibody-mediated blockade of the IFN receptor (IFNAR1). Each box plot represents the module score distribution from the full set of individual cells. The center line represents the median, the box limits represent the top and bottom quartiles, and the whiskers represent the minimum and maximum. **j**, Heat maps for each cell class, showing expression of the unique differentially expressed genes between *t*_0_ and *t*_0.25_ (see **d**) for control (*t*_0_), arthritis (*t*_0.25_), EndoS and IFN blockade. **k**, Bar plots showing the number of unique differentially expressed genes between *t*_0_ and *t*_0.25_ (see also **d** and **j**) and remaining differentially expressed genes after EndoS and IFN block treatments. nDE, number of differentially expressed genes.

**Extended Data Fig. 4:**
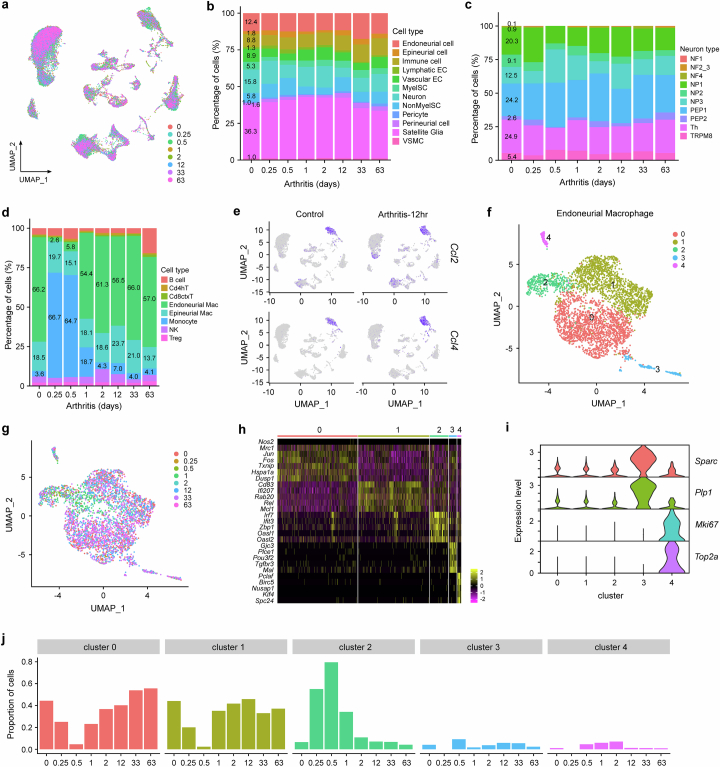
DRG neuronal and immune cells from autoantibody injected mice identified by sc-RNA seq. **a**, UMAP of the distribution of cell clusters (down sampled 5 000 cells from 86 052 cells) from autoantibody injected (0.25, 0.5, 1, 2, 12, 33, 63 days) and control DRG samples. **b**, Stacked bar plot of the cell type composition (percentage) at different timepoints of after autoantibody injection. **c**, Stacked bar plot of the neuron type composition (percentage) at different timepoints after autoantibody injection. **d**, Stacked bar plot of the composition of immune cells (percentage) at different timepoints after autoantibody injection. **e**, UMAP feature plots for *Ccl2* and *Ccl4* expression in DRGs (12 h) from control and autoantibody injected mice show local induced expression in immune cells, non-myelinating Schwann cells and endoneurial macrophages (9 500 cells from each sample). **f**, UMAP of the distribution of sub-clustering for endoneurial macrophage. **g**, UMAP of subtypes of endoneurial macrophages showing intermingled distribution of different macrophages from DRGs of control and autoantibody injected mice. **h**, Heatmap plot shows the top marker genes for each subtype of endoneurial macrophages. **i**, Stacked violin plot shows marker genes selectively expressed in clusters 3 and 4. **j**, Dynamic composition of sequenced endoneurial macrophage subtypes in the DRG at different timepoints after autoantibody injection.

To dissect molecular underpinnings and neuro–immune cross-talk, we performed DRG scRNA-seq at 6 and 12 h and days 1, 2, 12, 33 and 63 after vehicle or autoantibody treatment (Extended Data Fig. 3b–f and Supplementary Tables 1–4). We sequenced ~86,000 cells, including nerve sheath fibroblasts (perineurial cells, epineurial cells and endoneurial cells), endothelial and lymphatic endothelial cells, pericytes, vascular smooth muscle cells, satellite cells, nonmyelinating and myelinating Schwann cells, immune cells and neurons (Fig. 4a). All control cell types (time 0) appeared at all timepoints, and we saw no major shifts in gross cell-type proportions (Extended Data Fig. 4a,b). Higher-resolution immune cell analysis identified B cells, CD4^+^ and CD8^+^ T cells, natural killer (NK) cells, regulatory T cells, endoneurial and epineurial macrophages and monocytes (Fig. 4b); neurons clustered into known subtypes^10^ (Fig. 4c). Neuron subtype proportions remained stable (Extended Data Fig. 4c), but immune composition changed dramatically: monocytes increased ~20-fold at 6–12 h (from ~3.5% to ~65%) and returned to baseline by day 2, whereas endoneurial macrophages fell from ~65% to ~3% and recovered to ~60% in the same window (Extended Data Fig. 4d). *Ccl2* and *Ccl4* expression was rapidly upregulated in multiple non-neuronal and immune DRG cell types at 12 h (Extended Data Fig. 4e), consistent with CCL2/CCL5-dependent recruitment of CCR2/CCR5^+^ monocytes^29^.

Unbiased macrophage subtype analysis identified five subtypes and annotated cells based on marker gene expression^30^. Within 12 h, immune regulatory/resolution (*Mrc1*-Mac, cluster 0, *Ccr2*^+^) and activated/inflammatory (*Rel*-Mac, cluster 1, *Ccr2*^+^) macrophages polarized into proinflammatory IFN response macrophages (*Ccl2*/*Ccl5*-Mac, cluster 2), whereas *Sparc*-Mac (antigen-presenting, cluster 3) and *Mki67*-Mac (proliferating, cluster 4, *Ccr2*^+^) cells remained stable (Extended Data Fig. 4f–j). We interpret this as polarization toward *Ccl2*/*Ccl5*-Mac cells and turnover of endoneurial macrophages from a new monocyte pool during early arthralgia (12 h). Thus, DRG endoneurial macrophages are largely CCR2^+^ and likely derived from infiltrating monocytes^31^. Differential gene expression revealed abrupt changes at 12 h in macrophages and all other DRG cell types (Fig. 4d and Supplementary Tables 1–4).

**Extended Data Fig. 5:**
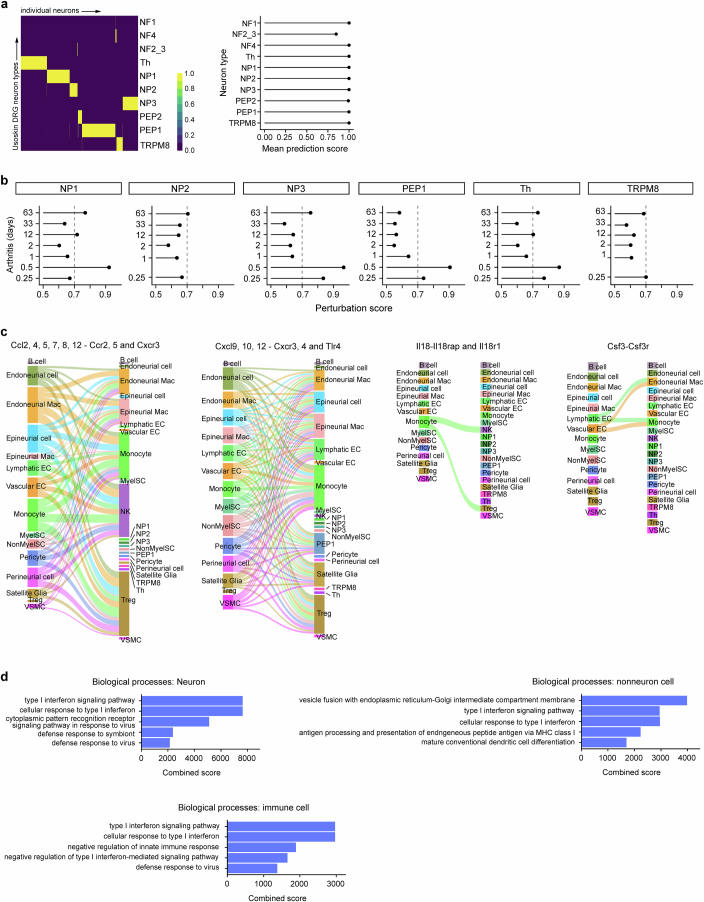
Transcriptional perturbation analysis for neuronal types and ligand-receptor interaction among DRG cell types. **a**, Heatmap (left) representing the predicted similarity score of individual neurons to the neuronal types according to Usoskin’s annotation through machine learning classifier. Mean prediction score (right) for different neuronal cell types. **b**, Perturbation score for DRG neuronal subtypes at different timepoints after injection of autoantibodies in mice. **c**, Ligand-receptor interactions induced at 12 h after autoantibody injection in mice. **d**, Representative biological processes of differentially expressed genes in neuron, nonneuron and immune cell types.

Machine learning-based neuron type assignment remained highly accurate (prediction scores near 1) under both control and autoantibody conditions (Extended Data Fig. 5a), indicating that autoantibodies do not alter cell-type-defining features. A classifier instead trained to distinguish experimental versus control cells identified NP1, NP3 and PEP1 sensory neurons as most perturbed, peaking at 12 h (Extended Data Fig. 5b). Thus, autoantibodies cause rapid, widespread molecular perturbations in sensory neurons without changing their core identity.

To explore cell–cell communication, we inferred ligand–receptor interactions using upregulated ligand genes at 12 h and scored interactions for enrichment relative to controls and random background (Supplementary Table 5). This generated an early arthritis interactome. Sankey plots of cytokine interactions showed CSF1 from endoneurial fibroblasts, vascular cells and satellite glia signaling to endoneurial macrophages and monocytes; IL-1β from endo- and epineurial macrophages targeting immune cells, vascular cells, fibroblasts and neurons; IL-6 from fibroblasts and vascular cells signaling broadly to all ganglion cell types and chemokines from non-neuronal and immune cells predominantly targeting immune populations (Fig. 4e and Extended Data Fig. 5c). Thus, even before visible joint inflammation, unbiased interaction analysis revealed a complex proinflammatory communication network driven by DRG non-neuronal and immune cells.

**Extended Data Fig. 6:**
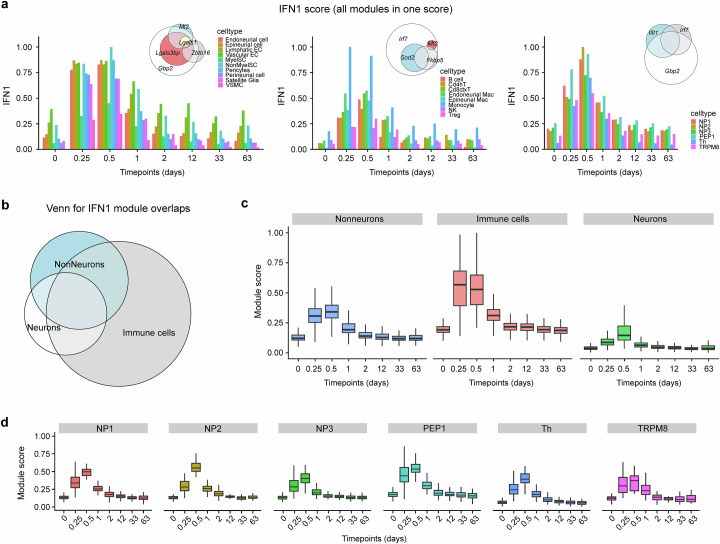
IFN1/antiviral module gene set enriched by GSEA and co-regulated gene set analysis for DRG cell types in mice with cartilage autoantibodies. **a**, IFN1/antiviral co-regulated genes module core for nonneuronal, immune and neuronal cell types at different timepoints after administration of autoantibodies. **b**, Venn diagram representing the overlapping of IFN1 module genes for nonneurons, neurons and immune cells. **c**, Boxplots of gene module scores for nonneuronal cells, immune cells and neurons by pseudobulk analysis at different timepoints after autoantibodies were delivered to the mice. **d**, Boxplots of gene module scores for individual neuronal clusters at different timepoints after autoantibodies were delivered to the mice. Boxplots represent the module score distribution from the full set of individual cells in (**c** and **d**). The center line represents the median; the box limits represent the upper and lower quartiles; the whiskers represent the minimum and maximum.

We next used Gene Ontology, hallmark gene set enrichment analysis (GSEA) and co-regulated gene set analysis on 12-h data. Across all cells combined and stratified into non-neuronal, immune and neuronal compartments, the highest-scoring Gene Ontology pathways involved type 1 IFN signaling and IFN responses (Fig. 4f and Extended Data Fig. 5d). Consistently, top GSEA pathways in non-neuronal, immune and neuronal cell types were IFNα response, with weaker enrichment for MYC, TNF and IL-6 signaling and other hallmarks (Fig. 4g and Supplementary Fig. 2). Gene network analysis identified several co-regulated type 1 IFN-related gene modules across cell types (Extended Data Fig. 6a,b), which we merged into a single type 1 IFN module. Module scores in pseudobulked non-neuronal cells, immune cells, neurons and individual neuronal subtypes revealed a rapid, transient induction of IFN-stimulated genes (ISGs) in all DRG cell types (Extended Data Fig. 6c,d). These included IFN signaling components, immunoproteasome/antigen presentation genes and antiviral effectors (Fig. 4h, neurons).

**Extended Data Fig. 7:**
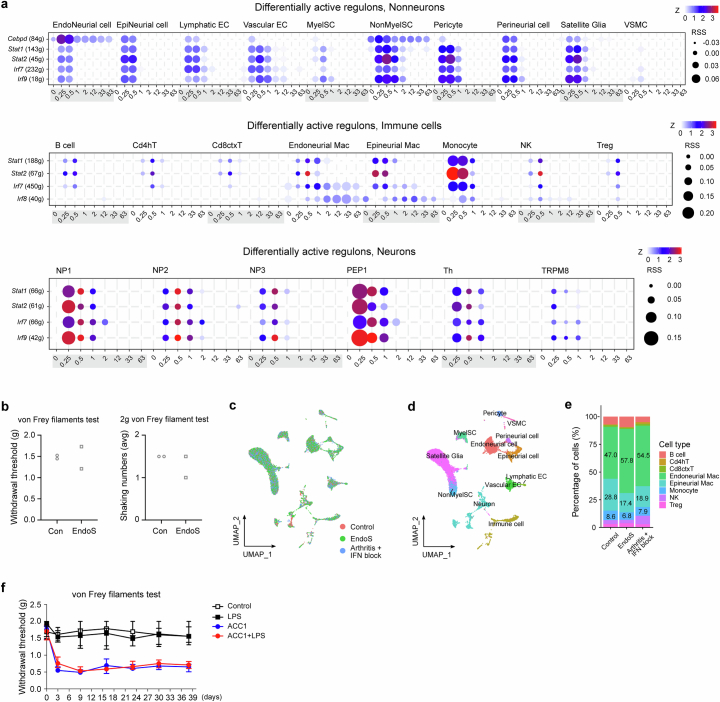
SCENIC analyses for gene regulatory networks in DRGs and blocking experiment. **a**, Dot plots representing enriched IFN-related gene regulons in nonneuronal cells, immune cells and neurons at different timepoints after administration of autoantibodies. **b**, Mechanical allodynia and hyperalgesia assayed in control and mice injected with EndoS treated autoantibodies (n = 2 per group). Pain-like behavioral tests of von Frey filaments (threshold) and 2 g von Frey filament (shaking numbers) show that EndoS treated cartilage autoantibody (EndoS) failed to initiate pain (12 h). **c**, UMAP of sequenced DRG cells showing intermingled distribution of cells from control, EndoS treated autoantibodies and autoantibodies (Arthritis) combined with in vivo IFN block (IFNAR1 antibody). **d**, UMAP of annotated DRG cell types from sequenced DRG samples in (**c**). **e**, Stacked bar plot of the composition of immune cells (percentage) from different DRG samples. **f**, Mechanical allodynia assessed following ACC1 antibody alone and ACC1 combined with LPS injection in C57BL/6 N mice (n = 6/group). Note no changes in control or LPS alone group. Data are expressed as median with interquartile range for (**f**). Source data

IFN1 receptor (IFNAR1/IFNAR2) activation phosphorylates STAT1 and STAT2 and recruits IRF9 to form ISGF3, which drives ISG expression^32^. We used SCENIC to infer master transcription factor regulons across all cell types and timepoints (Extended Data Fig. 7a and Supplementary Table 6). We identified 27 regulons; four neuron-specific regulons increased during the first 24 h after autoantibody treatment: IRF7, IRF9, STAT1 and STAT2 (42–66 target genes). In immune cells, autoantibody-associated regulons included IRF7, IRF8, STAT1 and STAT2 (40–450 genes) and in non-neuronal cells IRF7, IRF9, STAT1 and STAT2 (18–242 genes). Some non-neuronal cells also showed CEBPD regulon, consistent with IL-1β-, TNF- and IL-6-driven inflammatory transcription^33^, matching ligand–receptor results identifying non-neuronal cells and macrophages/monocytes as main proinflammatory sources (Fig. 4e and Extended Data Fig. 5c).

Together, these analyses identify type 1 IFN as the dominant driver of early transcriptional changes in DRG cells after autoantibody exposure. These data suggest that IFNα/IFNβ activation of IFNAR and ISGF3, rather than cytosolic nucleic acid sensors, induces ISGs.

To exclude contamination of the antibody cocktail with nucleic acids or pathogens, we inactivated Fc effector function by cleaving the Fc N-linked glycan at position 297, required for antibody binding to Fcγ receptors and effector function, using EndoS from *Streptococcus pyogenes*^34,35^. EndoS-treated, repurified cartilage-binding antibodies delivered at the same dose no longer induced allodynia or hyperalgesia (Extended Data Fig. 7b). DRG scRNA-seq at 12 h revealed that EndoS-treated autoantibodies did not affect the identification of all DRG cell types but failed to induce ISG module activation, monocyte infiltration or the autoantibody-associated differential gene expression program (Fig. 4i–k and Extended Data Fig. 7c–e). Thus, functional Fcγ receptor-binding autoantibodies are required for gene regulation and hyperalgesia.

Consistently, the collagen type 2-reactive autoantibody ACC1 alone, without LPS, sufficed to increase pain behavior, whereas LPS alone had no effect (Extended Data Fig. 7f), confirming that collagen-reactive autoantibodies are causal. To test whether type 1 IFNs directly mediate gene expression changes, we pretreated mice with an antibody that blocks IFNAR1 (MAR1-5A3), administered arthritogenic autoantibodies and performed DRG scRNA-seq at 12 h. IFNAR1 blockade prevented monocyte infiltration, ISG induction and regulon activation and normalized differential gene expression (Fig. 4i–k and Extended Data Fig. 7c–e). These results show that cartilage-binding autoantibodies, through Fc-glycan-dependent immune complex formation, trigger type 1 IFN release and IFNAR activation, leading to ISGF3-driven ISG expression. Thus, blocking type 1 IFN fully reverses the early inflammatory DRG state, identifying IFN1 as the dominant proinflammatory factor driving the autoantibody-induced inflammatory reaction.

### Persistent transcriptional alterations in endoneurial macrophages

**Extended Data Fig. 8:**
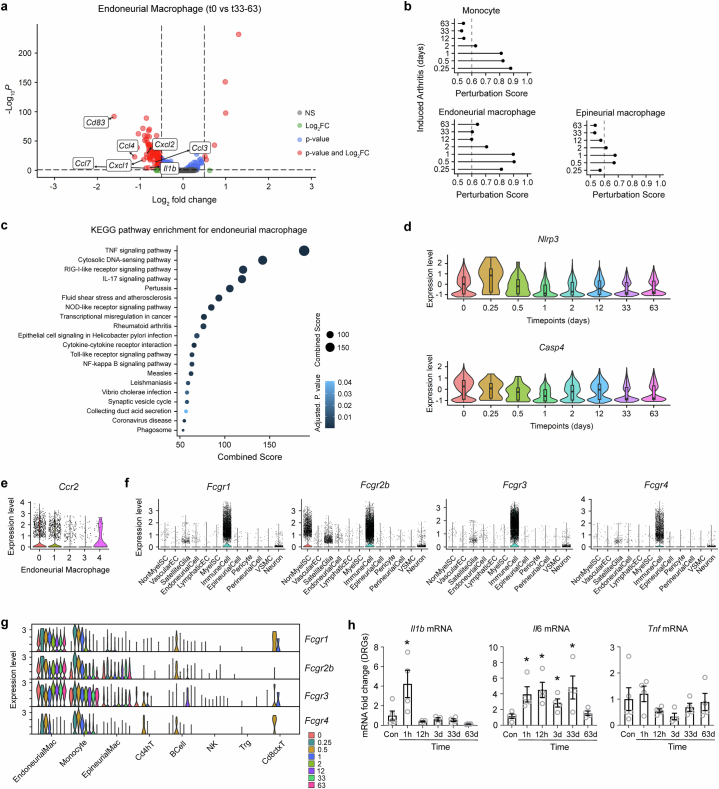
Differential gene expression analysis for endoneurial macrophages in DRGs of autoantibody injected mice. **a**, Volcano plot of differentially regulated genes from endoneurial macrophages at late (post-inflammatory) phase of arthritis with down-regulated cytokine genes highlighted. Statistical significance was calculated using two-sided Wilcoxon test adjusted for multiple comparisons using the Bonferroni correction. **b**, Transcriptional perturbation score for non-neuronal DRG cell types (monocytes, endoneurial and epineurial macrophages) at different timepoints after injection of cartilage autoantibodies. **c**, KEGG pathways of differentially expressed genes in endoneurial macrophages at the post-inflammatory phase of arthritis. Statistical significance was calculated using a two-sided Fisher exact test corrected for multiple comparisons using the Benjamini-Hochberg method The combined score was calculated by multiplying the log of the p-value by the z-score of the deviation from the expected rank. **d**, Gene expression level of *Nlrp3* and *Casp4* in DRGs from autoantibody-injected mice all timepoints. **e**, Expression of *Ccr2* in the different endoneurial macrophage types (all timepoints combined). **f**, Expression level of *Fcgr* genes in the different immune cell types across timepoints. Note expressions are mainly in endoneurial macrophages and monocytes. **g**, Violins of Fcgr (*Fcgr1-4*) receptor expression in DRG cell types across timepoints. Note abundant expression in immune cells. **h**, qPCR of indicated cytokines in DRG tissue at different timepoints after autoantibody administration (n = 4/5 for controls and n = 4/timepoint for arthritis). Data for qPCR were expressed as mean with s.e.m. and analyzed with two-tailed unpaired t test. * indicates *P* < 0.05. The exact *P* values are listed in the source data. Source data

Because pain behavior preceded arthritis onset and persisted after arthritis resolved, we considered the transient inflammatory reaction unlikely to fully explain arthritis pain. The only DRG cell type showing persistent gene expression changes across all timepoints was endoneurial macrophages (Fig. 4d and Extended Data Fig. 8a). A machine learning module trained to identify experimental versus control cells reliably identified endoneurial macrophages from autoantibody-treated mice up to 63 days, whereas epineurial macrophages and monocytes were perturbed only within the first day (Extended Data Fig. 8b). Persistent changes in endoneurial macrophages were few, mostly downregulated and distinct from early 12-h responses. KEGG analysis highlighted TNF signaling pathway alterations, suggesting sustained suppression of some proinflammatory features during active joint inflammation (day 12) and after remission (days 33 and 63; Extended Data Fig. 8c and Supplementary Table 7).

**Fig. 5:**
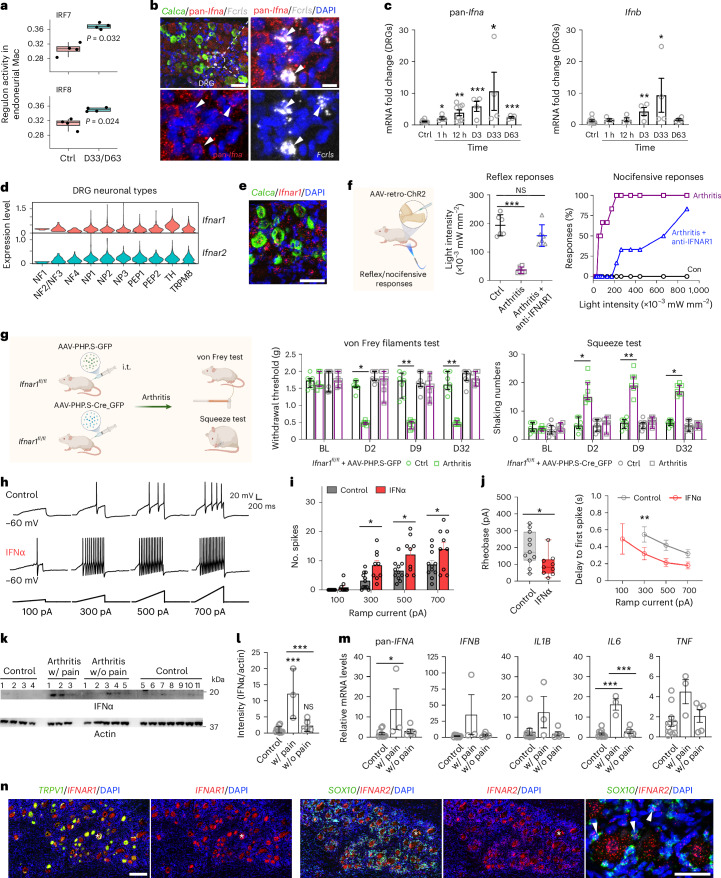
Sustained IFN signaling drives arthritis pain behavior. **a**, Box plots showing IRF7 and IRF8 regulon activity in endoneurial macrophages. Regulon activity was scaled between 0 and 1 for each regulon. *P* values were derived using a permutation test with 1,000 random label assignments. **b**, Representative RNAscope images of triple labeling for pan-*Ifna*, *Calca* and *Fcrls* mRNAs in mouse DRGs (*n* = 5 mice). Scale bars, 50 µm (left) and 10 µm (right). **c**, Expression changes in *Ifna* and *Ifnb* after autoantibody injection in mouse DRGs as determined by qPCR (*n* = 9 mice for control, 8 mice for 12 h and 4 mice for each of the other groups). Ctrl, control. **d**, Expression of *Ifnar1* and *Ifnar2* from the mouse DRG scRNA-seq dataset. **e**, Representative RNAscope image of *Ifnar1* expression in DRG neurons, with some colocalization with the peptidergic neuronal marker *Calca* (*n* = 5 mice). Scale bar, 50 µm. **f**, Light threshold of withdrawal and nocifensive responses before and after treatment with anti-IFNAR1 (i.p., 40 mg per kg (body weight)) in mice with arthritis that received intra-articularly injected AAV-retro-ChR2 virus (*n* = 6). **g**, Neuronal knockout of *Ifnar1* through AAV-PHP.s-Cre virus in *Ifnar1*^*fl/fl*^ mice (*n* = 7 per group). i.t., intrathecal. **h**, Current clamp responses of cultured mouse DRG neurons to IFNα (*n* = 11 for control, *n* = 9 for treatment). **i**, Numbers of spikes from recorded DRG neurons in **h**. **j**, Current clamp recording parameters of rheobase and the delay to first spike from **h**. **k**, IFNα expression in human DRGs from healthy individuals (*n* = 11) and individuals with arthritis with (w/) pain (*n* = 3) or without (w/o) pain (*n* = 5). **l**, Quantification of IFNα expression in **k**. **m**, Fold changes in cytokine mRNA expression in human DRGs from donors as in **k**. **n**, Representative RNAscope images of *IFNAR1* and *IFNAR2* mRNA expression in DRGs from healthy female donors (*n* = 2). Asterisks indicate examples of neurons with lipofuscin. Scale bars, 200 µm (left) and 50 µm (right). Cartoons in **f** and **g** were created in BioRender; Fatt, M. https://biorender.com/2wkmnd5 (2026). Data are expressed as mean ± s.e.m. (**c**,**i**,**j**,**m**), median with interquartile range (**g**), mean ± s.d. (**l**) or boxes with whiskers (minimum to maximum show all points) (**a**,**j**). For the box plots, the center line represents the median, the box limits represent the top and bottom quartiles, and the whiskers represent the minimum and maximum. qPCR data (**c**) were analyzed with a two-tailed unpaired *t*-test. Behavioral data (**g**) were analyzed by Kruskal–Wallis test followed by a Dunn’s multiple comparisons test. Optogenetic reflex (**f**), western blotting (**l**) and qPCR (**m**) data were analyzed by one-way ANOVA, followed by a Tukey’s or Bonferroni’s multiple comparisons test. Patch clamp analysis (**i**,**j**) was performed with a two-way ANOVA followed by a Šídák’s multiple comparisons test, while rheobase data were analyzed by two-tailed unpaired *t*-test. Arrowheads indicate colocalization in **b** and **n**. *P < 0.05, **P < 0.01 and ***P < 0.001. The exact and adjusted *P* values are listed in the source data. Source data

Priming of the NLRP3 inflammasome requires upregulation of NLRP3, CASP4 and IL-1β expression via NF-κB signaling^10,36^. Endoneurial macrophages showed sustained downregulation of *Nlrp3* and *Casp4* expression at all timepoints except day 1, as well as decreased *Il1b* and chemokine transcript expression at later times (Extended Data Fig. 8a,d), including reduced NF-κB components. CCR2^+^ endoneurial macrophage subtypes (*Mrc1*-Mac, *Rel*-Mac) associate with fenestrated capillary endothelial cells^37^ (Extended Data Fig. 8e). Macrophages expressed activating Fcγ receptors *Fcgr1* and *Fcgr3*, whereas Schwann cells expressed low levels of inhibitory *Fcgr2b* (Extended Data Fig. 8f,g). The persistent alteration in macrophage gene expression was associated with activity in the gene regulatory networks (that is, regulons) of IRF7 and IRF8 (Fig. 5a, Extended Data Fig. 7a and Supplementary Table 6). IRF7 directly transactivates type 1 IFNs^38^, suggesting that type 1 IFNs may remain important during and after active arthritis.

### Persistent type 1 IFN expression and its effects on neuronal excitability

We could not detect *Ifna* transcripts by scRNA-seq, so we used RNA in situ hybridization with probes targeting conserved *Ifna* regions. We observed *Ifna* expression in endoneurial macrophages and neurons (Fig. 5b). Quantitative PCR (qPCR) for all 14 *Ifna* and *Ifnb* transcripts in the mouse DRG showed sustained upregulation of type 1 IFN transcripts throughout all disease stages after autoantibody-induced arthritis (Fig. 5c). *Ifnar1* and *Ifnar2* mRNAs were present in all DRG neuron subtypes (Fig. 5d), and in situ hybridization confirmed broad *Ifnar1* expression including *Calca*^+^ neurons (Fig. 5e). qPCR for *Il1b*, *Il6* and *Tnf* mRNAs revealed increased *Il6* expression from 1 h to day 33, transient *Il1b* increase at 1 h and no changes in *Tnf* expression (Extended Data Fig. 8h). Thus, type 1 IFNs are the only cytokines consistently increased in the DRG into the postinflammatory phase.

We then asked whether type 1 IFNs could account for hyperexcitability of joint afferents. We expressed ChR2 in joint afferents via AAV-retro-ChR2 injection into the ankle and induced arthritis with autoantibodies, and at day 32 we administered anti-IFNAR1 and measured withdrawal thresholds and nocifensive responses to blue light activation of articular afferents before and 1 h after treatment (Fig. 5f). As observed before, naive mice showed withdrawal but no nocifensive responses even at high light intensities, whereas autoantibody-treated mice displayed decreased reflex thresholds and robust nocifensive behavior (Fig. 5f and Extended Data Fig. 2g). IFNAR1 blockade (40 mg per kg (body weight), i.p.) normalized reflex thresholds to naive levels and largely reversed nocifensive responses (Fig. 5f).

**Extended Data Fig. 9:**
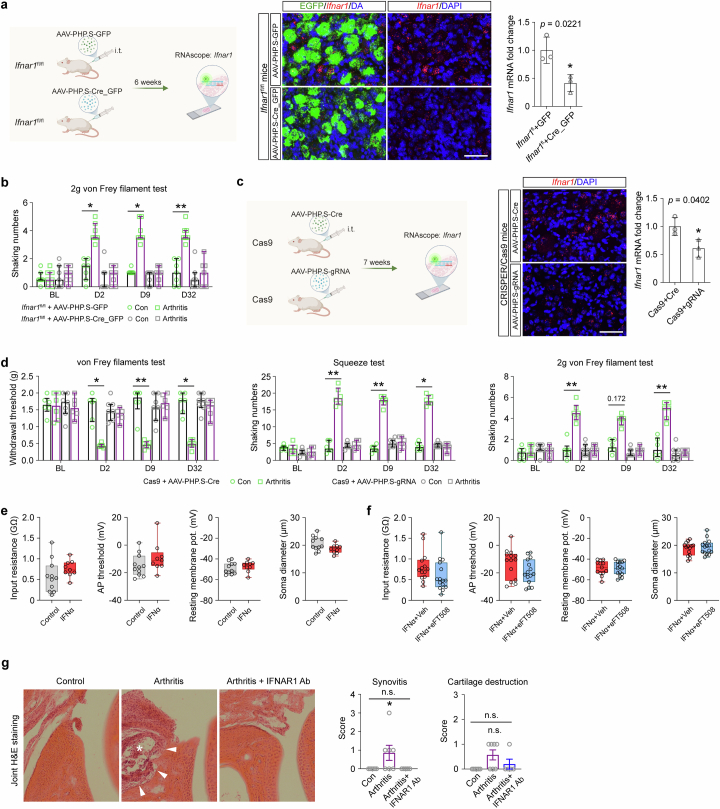
Knockout of Ifnar1 in sensory neurons prevents autoantibody-induced pain behaviour. **a**, Knockout of *Ifnar1* gene was performed by intrathecal injection with AAV-PHP.S-CAG-Cre_EGFP virus into Ifnar1^fl/fl^ mice (efficiency 41.5±15.0%, n = 3/group). **b**, Autoantibody-induced mechanical allodynia in Ifnar1^fl/fl^ mice intrathecally injected with control virus (AAV-PHP.S-GFP), but not in Ifnar1^fl/fl^ mice with AAV-PHP.S-Cre_GFP virus (n = 7/group). **c**, *Ifnar1* gene knockout by a CRISPR-Cas9 strategy, where the efficiency was 60.9±15.8% compared to controls (n = 3/group). **d**, Autoantibody-induced mechanical hypersensitivity and joint pain only showed in Cas9 mice intrathecally received AAV-PHP.s-Cre but not CRISPR-gRNA-Ifnar1 AAV virus (n = 6/group). **e**, Current clamp recording parameters of input resistance, action potential threshold, resting membrane potential and soma diameter for cultured DRG neurons with no treatment (Control, n = 11) and IFNα stimulation (IFNα, 300 U/ mL, n = 9). **f**, The same parameter analyses as in (**e**) from cultured DRG neurons treated with IFNα + vehicle (DMSO, n = 14) or IFNα + eFT508 (MNK1/2 inhibitor, n = 15). **g**, Histopathological images and scoring of ankle joint from control, arthritis and IFNAR1 Ab repeated treated arthritis Balb/c mice (d60). The arthritic joint showed pannus formation (synovitis, asterisk) with bone erosion (arrowhead), (n = 6 for control, 7 for arthritis and 5 for treatment group). Cartoons in (**a** and **c**) were created in BioRender. Fatt, M. (2026) https://BioRender.com/2wkmnd5. Data are expressed as mean with s.d. in (**a** and **c**), median with interquartile range in (**b** and **d**), median with range (box with whiskers) in (**e** and **f**) or mean with s.e.m. in (**g**). For boxplots, the center line represents the median; the box limits represent the upper and lower quartiles; the whiskers represent the minimum and maximum. Knockout efficiency and patch-clamp results were analyzed with two-tailed unpaired *t*-test in (**a**, **c**, **e** and **f**). Behavioral data in (**b**) and (**d**) were analyzed by Kruskal-Wallis test followed by Dunn’s multiple comparisons test. Histological analysis was performed with Kruskal-Wallis test followed by Dunn’s multiple comparisons test. * indicates *P* < 0.05, ** indicates *P* < 0.01, *** indicates *P* < 0.001 and n.s. means no significance. The exact and adjusted *P* values are listed in the source data. Source data

To test whether type 1 IFNs act directly on sensory neurons, we deleted *Ifnar1* selectively in DRG neurons using two strategies: AAV-PHP.s-Cre injection in *Ifnar1*^*fl/fl*^ mice and AAV-PHP.s-guide RNAs (gRNAs) in R26-Cas9 mice. Both approaches efficiently deleted *Ifnar1* in sensory neurons (Extended Data Fig. 9a,c). In both models, autoantibody-induced hyperalgesia, allodynia and joint pain behavior depended on neuronal IFNAR1; *Ifnar1* deletion reversed all pain phenotypes (Fig. 5g and Extended Data Fig. 9b,d). Thus, IFN1–IFNAR1/IFNAR2 signaling in sensory neurons drives arthritis pain.

Patch clamp recordings of small/medium DRG neurons exposed to IFNα for 1 h showed increased excitability, specifically more action potentials, reduced rheobase and shorter latency to first spike (Fig. 5h–j and Extended Data Fig. 9e), consistent with previous work^8^ and supporting increased intrinsic excitability as a mechanism for sensory dysfunction.

To translate these findings to humans, we analyzed DRG tissue from donors with RA and joint pain, donors with RA without joint pain and healthy control donors (Supplementary Table 8). IFNα protein levels were higher in DRG tissue from individuals with RA with pain than in the other groups (Fig. 5k,l). qPCR for all 13 *IFNΑ* (conserved primers) transcripts and *IFNΒ* also showed increased type 1 IFN mRNA transcript expression in DRG tissue from individuals with RA with pain compared to those with RA without pain and control healthy individuals (Fig. 5m). *IL6* mRNA expression was likewise increased in DRG tissue from individuals with RA with pain (Fig. 5m). In situ hybridization showed *IFNAR1* expression in *TRPV1*^+^ and *TRPV1*^−^ sensory neurons; *IFNAR2* was expressed broadly in neurons and in *SOX10*^+^ satellite glia (Fig. 5n and Supplementary Fig. 3). Thus, IFN receptors are expressed by sensory neurons in mice and humans, and both the mouse arthritis model and individuals with RA with pain show increased DRG type 1 IFN expression.

### A persistent ‘non-ISGF3’ IFN1–MNK1/MNK2–eIF4E pathway causes sensory neuron hyperexcitability

**Fig. 6:**
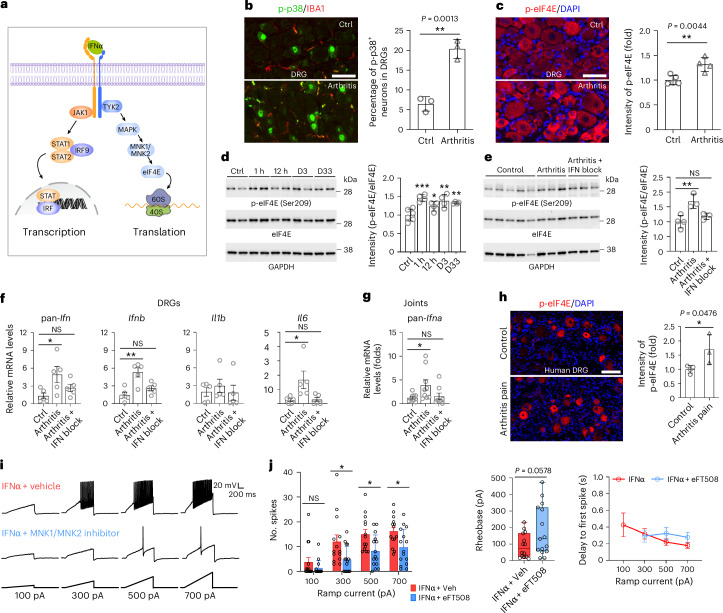
A persistent type 1 IFN-induced MNK1/MNK2–eIF4E signaling pathway in arthritis causes neuronal hyperexcitibility. **a**, Illustration of transcriptional and translational pathways activated by type 1 IFNs. **b**, Representative immunohistochemistry images of phosphorylated p38 (p-p38) and IBA1 in the DRG, where the proportion of p-p38^+^ neurons was quantified (*n* = 3 per group). **c**, Representative immunohistochemistry images of p-eIF4E (Ser209) in control DRG tissue and DRG tissue from the arthritis group; intensity of p-eIF4E in neurons was quantified (*n* = 4 per group). **d**, Phosphorylation of eIF4E (Ser209) in the DRG in mice with arthritis (normalized to eIF4E). Each time point contains four mice, except the control group (*n* = 6). **e**, Effect on phosphorylation of eIF4E by i.p. injection of anti-IFNAR1 (IFN block) in mice with arthritis; *n* = 4 in each group. The fifth control sample was not included for quantification. **f**, Fold changes in *Ifna*, *Ifnb*, *Il1b* and *Il6* expression in mouse DRGs from control, arthritis and treatment groups (*n* = 5 per group). **g**, *Ifna* gene expression in ankle joint synovia from mice with arthritis treated with anti-IFNAR1 (day 33; *n* = 8 or 9 per group). **h**, Representative immunohistochemistry images of p-eIF4E (Ser209) in human DRGs from healthy donors (*n* = 4) and individuals with arthritis with pain (*n* = 3). **i**, Current clamp recordings of cultured mouse DRG neurons exposed to IFNα and the MNK1/MNK2 inhibitor eFT508. **j**, Plots for number of spikes, rheobase and delay to first spike recorded from IFNα-stimulated DRG neurons treated with MNK1/MNK2 inhibitor (*n* = 14 for vehicle (Veh), *n* = 15 for eFT508). For the box plots, the center line represents the median, the box limits represent the top and bottom quartiles, and the whiskers represent the minimum and maximum. Scale bars, 50 µm (**b**,**c**) and 100 µm (**h**). Data are expressed as mean ± s.d. (**b**–**e**,**h**) or mean ± s.e.m. (**f**,**g**,**j**). Rheobase data are expressed as a box with whiskers (minimum to maximum show all points) (**j**). Immunohistochemistry and rheobase data were analyzed by two-tailed unpaired *t*-test, and qPCR and western blotting data were analyzed by one-way ANOVA followed by a Tukey’s multiple comparisons test. Patch clamp analysis was performed with a two-way ANOVA followed by a Šídák’s multiple comparisons test. *P < 0.05, **P < 0.01 and ***P < 0.001. The exact and adjusted *P* values are listed in the source data. Source data

Type 1 IFNs typically induce ISGs via ISGF3, but this response is transient and self-limiting through feedback inhibition^39^. The absence of ongoing ISG expression during inflammatory and postinflammatory phases despite continuous type 1 IFN presence suggested an alternative persistent signaling mechanism underlying arthritis pain. Non-ISGF3 type 1 IFN signaling can engage p38 MAPK, MNK1/MNK2 and eIF4E to modulate translation^40^ (Fig. 6a).

We examined MNK1/MNK2–eIF4E pathway activation in arthritis in vivo by quantifying phosphorylation. DRG neurons from day 33 autoantibody-treated mice showed increased p-p38 compared to naive controls (Fig. 6b). eIF4E Ser209 phosphorylation (p-eIF4E), a direct readout of MNK1/MNK2 activity^40^, was increased in DRG neurons from autoantibody-treated mice (Fig. 6c). Time course analysis at 1 h, 12 h, day 3 and day 33 showed increased p-eIF4E at all timepoints (Fig. 6d and Supplementary Fig. 4a). IFNAR1 blockade on day 33 reversed p-eIF4E elevation (Fig. 6e and Supplementary Fig. 4b), demonstrating that type 1 IFN drives sustained MNK–eIF4E activation.

IL-6, like type 1 IFNs, can signal through MNK1/MNK2 and sensitize sensory neurons^41^, so both type 1 IFNs and IL-6 might converge on MNK1/MNK2. We tested whether chronic IL-6 elevation depended on type 1 IFNs. IFNAR1 blockade at day 33 reversed increased IL-6, IFNα and IFNβ expression in the DRG of autoantibody-treated mice (Fig. 6f), indicating type 1 IFN dominance. In synovial tissue, *Ifna* was increased, whereas *Ifnb*, *Il1b* and *Il6* were undetectable and *Tnf* was unchanged; IFNAR1 blockade also reversed *Ifna* elevation in the synovium (Fig. 6g). Phosphorylated eIF4E (Ser209) was also increased in DRG neurons from individuals with RA with pain compared to in healthy control individuals (Fig. 6h). We conclude that type 1 IFN engages a persistent MNK1/MNK2–eIF4E signaling pathway in DRG neurons.

To test whether IFNα-induced hyperexcitability depends on MNK–eIF4E, we performed patch clamp recordings of small DRG neurons perfused with IFNα with or without the MNK1/MNK2 inhibitor eFT508. MNK1/MNK2 inhibition normalized activation thresholds and prevented IFNα-induced increases in firing, reduced rheobase and shortened latency (Fig. 6i,j and Extended Data Fig. 9f). These results indicate that type 1 IFNs engage MNK1/MNK2 to cause sensory neuron hyperexcitability.

### Inhibition of type 1 IFN–MNK1/MNK2–eIF4E signaling reverses pain behavior

**Fig. 7:**
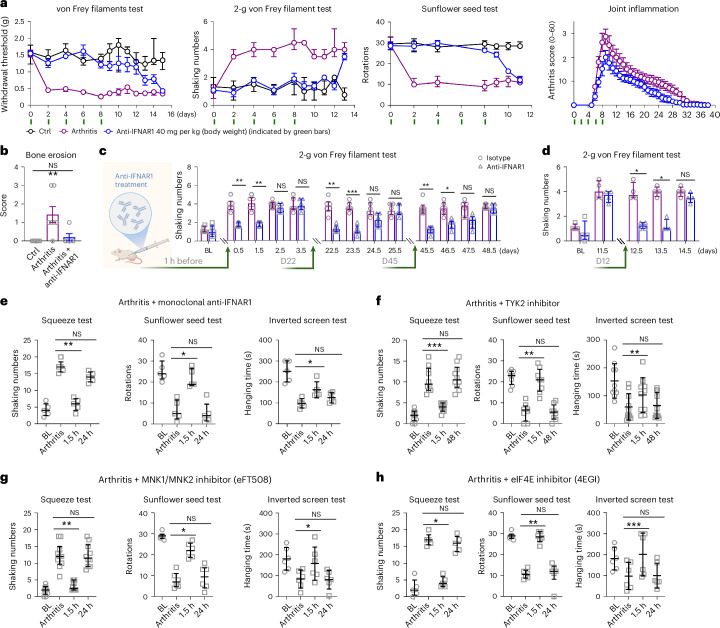
Inhibition of IFN signaling restores limb function and reverses pain in arthritis. **a**, Mechanical allodynia, hyperalgesia and dexterity in mice with arthritis with repetitive anti-IFNAR1 (days 0–8, i.p., 40 mg per kg (body weight)) treatment in BALB/c mice (male mice, *n* = 6 for control, 7 for arthritis and 5 for the treatment group). **b**, Bone erosion analysis of hematoxylin and eosin (H&E) staining in ankle joints from mice in **a** at day 60. **c**, Systemic administration of anti-IFNAR1 (40 mg per kg (body weight), i.p.) 1 h before cartilage autoantibody injection prevented mechanical hyperalgesia (2-g von Frey pricking pain behavior), and administration on days 22 and 45 reversed established hyperalgesia compared to the isotype IgG1 group (*n* = 6 per group). The cartoon was created in BioRender. Fatt, M. https://biorender.com/2wkmnd5 (2026). **d**, Anti-IFNAR1 administration on day 12 and its effect on mechanical hyperalgesia in mice with arthritis with peak inflammation (*n* = 4 per group). Arrows indicate the time of anti-IFNAR1 injection in (**c**,**d**). **e**, Effect of anti-IFNAR1 administration (i.p., 40 mg per kg (body weight)) on chronic joint pain behavior and impairment of dexterity and limb function in the squeeze, sunflower seed and inverted screen tests (C57BL/6N, *n* = 5). **f**, Effect of oral administration of TYK2 inhibitor (15 mg per kg (body weight)) on sensory deficits associated with arthritis (*n* = 8 for sunflower seed test; *n* = 10 squeeze and inverted screen tests). **g**, Effect of MNK1/MNK2 inhibitor (i.p., eFT508, 1 mg per kg (body weight)) on sensory deficits associated with arthritis (*n* = 10 for squeeze and *n* = 6 for sunflower seed and inverted screen tests). **h**, Effect of eIF4E inhibitor (i.p., 4EGI, 15 mg per kg (body weight)) on sensory deficits associated with arthritis (*n* = 6). All graphs show behavior before inducing arthritis (BL), at the postinflammatory phase following antibody-induced arthritis (arthritis), at the postinflammatory phase of arthritis 1.5 h after administration of compounds (1.5 h) and after washout of compounds (24 h or 48 h). Data are expressed as mean ± s.e.m. for joint inflammation (**a**) and joint histology (**b**) or median with interquartile range for behavioral data. Scores of bone erosion (**b**), the 2-g von Frey filament test (**c**,**d**) and the squeeze and sunflower seed tests (**e**–**h**) were analyzed using a Kruskal–Wallis test followed by a Dunn’s multiple comparisons test. The inverted screen test in **e**–**h** was analyzed with a repeated measures one-way ANOVA followed by a Bonferroni’s multiple comparisons test. *P < 0.05, **P < 0.01 and ***P < 0.001. Adjusted *P* values are listed in the source data. Source data

If type 1 IFN–MNK1/MNK2–eIF4E signaling drives arthritis pain, pathway inhibition in vivo should reverse it. We tested IFNAR1 blockade in various treatment paradigms. Preventive anti-IFNAR1 (before autoantibody treatment and then every other day to day 8) in BALB/c mice prevented allodynia, hyperalgesia and dexterity loss without altering arthritis scores (Fig. 7a) and reversed bone erosion (Fig. 7b and Extended Data Fig. 9g). After stopping treatment on day 8, nociceptive and motor deficits gradually reappeared, matching untreated mice with arthritis within days, consistent with ongoing type 1 IFN signaling.

**Extended Data Fig. 10:**
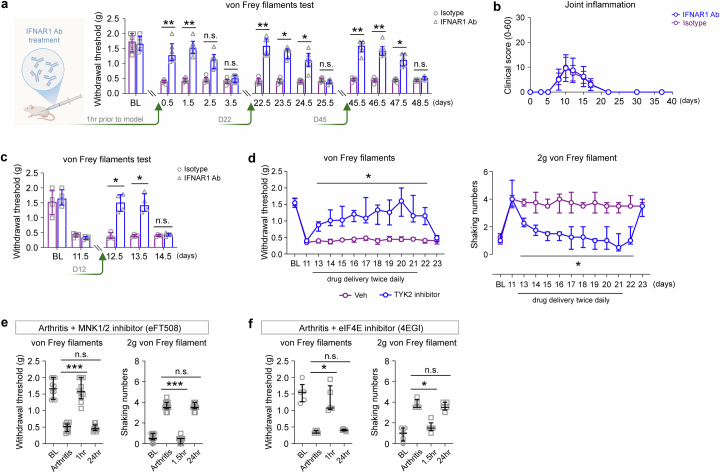
Sustained interferon signaling drives pain in arthritis. **a**, Systemic administration of IFNAR1 Ab (40 mg/kg, i.p.) one hour prior to cartilage autoantibody injection prevented mechanical allodynia (von Frey filaments) and administration on day 22 and day 45 reversed established allodynia and hyperalgesia compared to isotype IgG1 group (n = 6 per group). Cartoon was created in BioRender. Fatt, M. (2026) https://BioRender.com/2wkmnd5. **b**, Effects of treatment of IFNAR1 Ab (40 mg/kg, i.p.) as compared to isotype IgG1 administered prior to cartilage autoantibodies on joint inflammation represented as clinical score (n = 5 for isotype IgG1, 4 for IFNAR1 Ab). **c**, IFNAR1 Ab administration on day 12 reversed mechanical allodynia in arthritis mice with peak inflammation (n = 4 per group). **d**, Reversal of arthritis-induced allodynia and hyperalgesia by oral administrations of TYK2 inhibitor (Deucravacitinib, 15 mg/kg, twice daily, day13-day21, n = 5 or 6 per group). **e**, MNK1/2 inhibitor (eFT508, i.p., 1 mg/kg) blocked arthritis-induced allodynia and hyperalgesia in the post-inflammatory phase (n = 10 per group, BL, behavioral response prior to administration of cartilage antibodies; Arthritis, mice at the post-inflammatory phase after cartilage autoantibody administration. **f**, eIF4E inhibitor (4EGI, i.p., 15 mg/kg) reversed allodynia and hyperalgesia in the post-inflammatory phase of arthritis (n = 5 per group). Data are expressed as mean with s.e.m. in (**b**) or median with interquartile range for other behavioral tests in (**a** and **c**-**f**). Behavioral data in (**a**, **c**, **e** and **f**) were analyzed with Kruskal-Wallis test followed by Dunn’s multiple comparisons test for each drug delivery session. Data in (**d**) were analyzed with multiple Mann-Whitney test and corrected multiple comparisons using the Holm-Šídák method with alpha = 0.05. * indicates *P* < 0.05, ** indicates *P* < 0.01, *** indicates *P* < 0.001 and n.s. means no significance. The adjusted *P* values are listed in the source data. Source data

We next tested single-dose IFNAR1 blockade at different disease phases. A single IFNAR1 dose before autoantibody treatment transiently prevented allodynia and mechanical hyperalgesia; by 2.5–3.5 days, these returned to untreated levels (Fig. 7c and Extended Data Fig. 10a), consistent with the need to dose every other day to maintain signaling inhibition^42^. The single dose did not affect arthritis scores (Extended Data Fig. 10b). IFNAR1 blockade in mice with established arthritis (day 22) and in the postinflammatory phase (day 45) reversed mechanical allodynia and hyperalgesia for ~2 days (Fig. 7c and Extended Data Fig. 10a). At peak joint swelling (day 12), single-dose IFNAR1 inhibition efficiently reversed allodynia and hyperalgesia (Fig. 7d and Extended Data Fig. 10c). Importantly, paw squeeze joint pain behavior, forepaw dexterity deficits and limb usage disability (inverted screen) also reversed in the postinflammatory remaining pain phase (Fig. 7e).

IFNAR signals via TYK2 (ref. ^39^). We therefore tested whether the TYK2 inhibitor deucravacitinib (used in psoriasis) could mimic IFNAR blockade. Daily deucravacitinib treatment for 9 days robustly reversed allodynia, hyperalgesia and joint pain behavior and improved dexterity and limb function, with effects observed after a few days of dosing (Fig. 7f and Extended Data Fig. 10d). Inhibiting MNK1/MNK2 with eFT508, or eIF4E with 4EGI, fully reversed allodynia, hyperalgesia and joint pain behavior and improved dexterity and limb usage after a single dose in mice with arthritis (Fig. 7g,h and Extended Data Fig. 10e,f). Collectively, these data show that sustained type 1 IFN signaling engages MNK1/MNK2–eIF4E to sensitize nociceptors and cause joint pain in arthritis.

## Discussion

Identifying the neuronal population and biological processes causing arthritis pain is essential for new analgesic strategies. We combined mouse genetics, optogenetics, large-scale transcriptomics, computational biology, behavior, electrophysiology and biochemistry to build a molecular atlas of neuro–immune interactions underlying pain in arthritis across disease progression. We reveal acute and persistent inflammatory phases in the DRG. In the acute phase, autoantibodies engage immune, non-neuronal and neuronal DRG cell types, and locally produced cytokines/chemokines induce inflammatory gene networks in all ganglion cell types. In the persistent phase, cytokine expression and gene regulatory changes largely subside, except for IRF7/IRF8-dependent macrophage polarization. Our key conclusions are (1) discrete molecularly defined neuronal populations mediate arthritis pain; (2) type 1 IFNs are the dominant proinflammatory cytokines driving acute DRG inflammation and persistent pain; (3) acute type 1 IFN signaling induces ISGs, whereas a persistent non-ISGF3 pathway drives pain before, during and after arthritis; (4) persistent MNK1/MNK2–eIF4E signaling causes sensory neuron hyperexcitability; (5) conditional *Ifnar1* knockout in sensory neurons and pharmacological IFN1/MNK–eIF4E inhibition effectively alleviate joint pain and restore dexterity and paw function; and (6) in humans, local IFN production in the DRG is increased, type 1 IFN receptors are expressed by neurons, and there is increased MNK1/MNK2 activity in individuals with RA and pain, as in mice, suggesting a conserved mechanism.

### Sensory neurons mediating arthritis pain

Cartilage-binding autoantibodies induced arthralgia, allodynia, cold hyperalgesia and joint pain behavior, reduced dexterity and impaired limb use in mice. These behavioral deficits resemble emerging clinical insights into functional consequences of RA beyond inflammation and swelling^43^. Our initial idea was to infer the cell type responsible for pain in arthritis by identifying the hyperexcitable sensory neuron type but found that all nociceptor types were affected. The current view of some of the symptoms of RA such as stiffness, reduced grip strength and dexterous use of hands can be associated with inflammation and, hence, a consequence of joint inflammation and swelling^43^. This idea might be incomplete; stiffness and impairment in fine motor functions could be due to sensory–motor dysfunction across different populations of sensory neurons caused by IFNs because all neurons were sensitized and inhibiting IFN signaling reversed not only pain behavior but also dexterity and overall paw function seemingly independent of disease progression and remission. Therefore, IFN-induced functional alterations of sensory neuronal types beyond those causing joint pain is possible.

Sensory afferents are diverse and tuned to different pain qualities. Although CGRP^+^ and TRPV1^+^ afferents are widely considered mediators of joint pain, these markers define multiple A- and C-fiber types of sensory neurons. We found that silencing a defined C-fiber population (GFRA3^+^ PEP1 neurons) completely abolished pain in arthritis without altering baseline pain sensitivity to heat, cold or mechanical stimuli. Retrograde tracing showed that these neurons (especially PEP1.4^*Oprk1*^ subtype neurons) dominate joint innervation. There is a linear relationship between an increase in C-nociceptor impulses and the magnitude of reported pain rating in humans^44^. C-Nociceptor input can also increase and contribute to hyperalgesia by recruitment of so-called silent nociceptors (also called mechanically insensitive afferents). These normally reside in deep tissues and are relatively insensitive to mechanical stimuli in the absence of tissue injury but become mechanosensitive and spontaneously active when sensitized by inflammation^45^. GFRA3 is expressed in the silent nociceptors that acquire mechanosensitivity when exposed to the inflammatory mediator nerve growth factor^27,46^. These express CHRNA3^46^, which correspond to the PEP1.3a^*Adra2a*^ C-fiber subtype that were also found to innervate the joint. Even though silencing C-fiber GFRA3^+^ nociceptors abolished pain in arthritis, tracing of GFRA3^−^ A-fiber HTMR types PEP2.2^*Aldh1a1*^ and PEP3.2^*Bmpr1b*/*Prokr2*^ from joints indicates the presence of a fast component that potentially could contribute. PEP2.2^*Aldh1a1*^ neurons also innervate the skin, and their optogenetic activation induces reflexive withdrawal but not nocifensive behavior in mice^47^. Increased excitability of only articular afferents would have explained the neural basis for arthralgia. That we found increased excitability of all neuronal types while pain in RA is confined to peripheral joints is confounding. Furthermore, we found that optogenetic activation of the articular afferents in naive mice did not lead to pain behavior while activation of the same afferents in mice exposed to autoantibodies induced robust pain behavior. Thus, joint pain can be explained by sensitization processes locally in the DRG and in joints leading to increased excitability and altered function whereby non-nociceptive neurons signal nociception. The GFRA3^+^ neurons that belong to PEP1 C-heat/HTMRs are essential, and joint innervation points to the molecularly defined PEP1.4^*Oprk1*^ and PEP1.3a^*Adra2a*^ subtypes of PEP1 neurons.

### Persistent type 1 IFN–MNK1/MNK2–eIF4E signaling as the mechanism of pain

Arthritogenic autoantibodies in mice induced a rapid and broad inflammatory response in the DRG before joint pathology. All major DRG cell types, including neurons, glia, fibroblasts, vascular cells and immune cells, showed cytokine induction, inflammatory ligand–receptor networks and downstream transcriptional activation. Among these pathways, type 1 IFN signaling dominated. Type 1 IFN inhibition prevented nearly all inflammatory gene expression changes in the DRG. However, this transcriptional response was short lived and had fully resolved during ongoing pain. This temporal disconnect, together with the known antinociceptive effects of persistent STING–STAT1/IRF9-dependent ISG expression^48–51^, argues that ISGs themselves are not responsible for chronic pain in arthritis. Hence, activation of STING in DRG neurons triggers an IFNβ response and antinociception as well as promotes pain resolution in inflammation. This mechanism relies on constitutive activation of STING and concurrent IFNβ-induced signaling through STAT1/IRF9. STAT1/IRF9-induced ISG expression is responsible for reduced neuronal excitability where one key mechanism is gene expression of the voltage-gated potassium channel *Kcnip1* (K_v_4.3), which was among the ISGs, and K_v_4.3 was consistently confirmed to reduce the excitability of sensory neurons^51^. Instead, our evidence shows that persistent pain stems from an alternative IFN pathway (MNK1/MNK2–eIF4E). This pathway does not require continued ISG transcription and controls, among other things, cap-dependent translation. Type 1 IFN activated MNK1/MNK2 and phosphorylated eIF4E throughout arthritis, including postinflammatory phases. Blocking IFNAR1 reversed phosphorylated eIF4E and normalized the hyperexcitability of joint afferents. Patch clamp recordings confirmed that IFNα increased mouse sensory neuron excitability and that MNK1/MNK2 inhibition fully prevented IFN-evoked hyperexcitability, consistent with reported effects of type 1 IFNs on excitability of human DRG neurons^52^. These results show that chronic type 1 IFN signaling drives post-transcriptional remodeling of excitability via MNK1/MNK2–eIF4E, providing the mechanistic basis for sensory plasticity and persistent pain long after inflammatory resolution.

### Autoantibody-driven pathogenesis in the DRG

RA pathogenesis involves immune complex deposition in the synovium, activation of macrophages via Fcγ receptors and induction of TNF, IL-1β, IL-6 and degradative enzymes. Our data suggest that similar immune complex mechanisms occur in the DRG. The DRG contains perivascular neuro–immune units composed of macrophages, fibroblasts and pericytes surrounding PLVAP^+^ capillaries^10^ that extravasate circulating macromolecules^53^. CCR2^+^ endoneurial macrophages express Fcγ receptors, making them candidate sensors of autoantibody immune complexes leaking into the DRG. This could initiate macrophage polarization, local type 1 IFN production and persistent MNK–eIF4E signaling. Whether immune complexes form directly in DRG tissue or arrive preassembled from the synovium remains unresolved, but increased type 1 IFN was present in both the DRG and synovial tissue during postinflammatory phases. Because IRF7 and IRF8 were the only persistent regulons and were confined to endoneurial macrophages, these cells likely represent the chronic type 1 IFN source sustaining pathological signaling.

### Implications for RA pain treatment

Current pain management in RA is limited and often relies on analgesics that do not target underlying mechanisms. Broad-spectrum cytokine inhibition, including JAK inhibitors such as baricitinib, can reduce pain but carries the risk of severe adverse effects. Our data identify the persistent type 1 IFN–MNK1/MNK2–eIF4E pathway as a mechanistic driver of pain, independent of joint inflammation. In multiple treatment windows, inhibition of IFNAR, TYK2, MNK1/MNK2 or eIF4E reversed established allodynia, hyperalgesia, joint pain, dexterity impairment and limb dysfunction. Importantly, several inhibitors targeting this pathway (including anifrolumab, deucravacitinib and tomivosertib) already exist clinically or are in trials for other conditions, with known safety profiles. This lowers the barrier for therapeutic translation.

In summary, our results reveal an IFN signaling program as a mechanistic basis for pain in arthritis. Type 1 IFNs drive acute inflammatory transcriptional responses and via MNK1/MNK2–eIF4E a long-lasting sensory neuron plasticity, sensitizing defined C-HTMRs and generating arthralgia. The pathway is conserved in human RA and is pharmacologically reversible. These findings establish local IFN signaling as a root cause of persistent pain in arthritis and provide a targeted strategy for alleviating it.

## Methods

### Human samples and animals

Human DRG samples listed in Supplementary Table 8 were included in this study and were approved by the Swedish Ethical Review Authority (Etikprövningsmyndigheten). All animal experiments were performed in accordance with protocols approved by the Stockholm Ethical Committee for Animal Experiments (Stockholms Norra Djurförsöksetiska Nämnd, Sweden). Animals were provided with food and water ad libitum and were maintained on a 12-h light/12-h dark cycle. Wild-type C57BL/6N mice (adult, 8–9 weeks) were ordered from Charles River (Scanbur). Mouse strains targeting different subsets of neurons are included in Supplementary Table 9. All strains were backcrossed to C57BL/6N wild-type mice for at least three generations before being used for breeding.

For *TrkA*^CreERT2^ and *Gfra3*^CreERT2^ mice, tamoxifen (Sigma, T5648) was dissolved in corn oil (Sigma, 8267) at a concentration of 20 mg ml^−1^ and delivered by i.p. injection to postnatal day 14 pups once and then in adults for two consecutive days (140 mg per kg (body weight), both pups and adults). Control groups of test mice also received tamoxifen injections.

### Autoantibody-induced arthritis model

Arthritis was induced by i.v. injection of 6 mg of a mixture of four arthritogenic monoclonal antibodies (ACC1 (anti-citrullinated C1 epitope of collagen type II (COL2)), M2139 (anti-COL2), L10D9 (anti-collagen type XI) and 15A (anti-cartilage oligomeric matrix protein antibody)) on day 0, followed by 25 μg of LPS (055:B5, Sigma) i.p. on day 5 (ref. ^18^). Control mice received 150 μl of saline i.v. on day 0 and 100 μl of saline i.p. on day 5. LPS-treated mice were injected with 150 μl of saline i.v. on day 0 and 25 μg LPS i.p. on day 5. No changes in joint inflammation or pain-like behavior were observed in any strain of LPS-treated mice.

Visual joint inflammation was scored as previously described^54^. In brief, each inflamed (both swollen and redness) digital was given a score of 1 point, and if the dorsal side of the paw or wrist/ankle joint was inflamed, 2.5 points were given for moderate inflammation and 5 points for severe inflammation, resulting in a maximum of 15 for each limb and in total 60 per mouse.

### Light-induced response

A flexible optical fiber bundle monitored by a power controller (DC2200, Thorlabs) was used to activate ChR2, and the withdrawal reflex was elicited using a pulsing laser (470 nm, 10 Hz, 50 ms ON/OFF) with intensities from low to high and applied to the plantar surface of the hind paws. *Wnt1*-ChR2, *TrkA*^ChR2^, *Sst*^ChR2^, *Vglut3*^ChR2^, *Gfra3*^ChR2^ and *Mrgprd*^ChR2^ mice (*n* = 7–8) were habituated for 1 h on the mess floor, and a 20-s trial was conducted, alternating between the left and right hind paws with intervals of at least 10 min. The light threshold was determined as the lowest light power provoking a withdrawal response (for reflex) or nocifensive behavior like shaking, lifting, licking and guarding in one of the stimulated hind paws. The percentage of withdrawal reflex responding mice in different strains was reported. In all experiments, subthreshold light stimulations (0.2% lower intensity than threshold) were applied simultaneously combined with the following described behavioral tests.

### Behavioral tests

Mice were habituated on two occasions before assessment of baseline. After two baseline recordings performed on different days, the animals were randomly assigned to saline control and arthritis groups. Mechanical sensitivity was determined by assessment of paw withdrawal using von Frey filaments (Stoelting). A series of filaments with a logarithmically incremental stiffness of 0.04, 0.07, 0.16, 0.4, 0.6, 1.0 and 2.0 g was applied to the plantar surface of the hind paw and held for 3 s according to the up–down method, as previously described^55^. A brisk withdrawal of the paw was noted as a positive response. The 50% probability of withdrawal threshold (force of the von Frey hair to which an animal reacts to 50% of the presentations) was calculated as the threshold. To avoid any potential tissue damage, a cutoff value of 2.0 g was applied. The average withdrawal threshold of two hind paws was used.

To assess heat sensitivity, a radiant heat source (IITC) was aimed at the plantar surface of the hind paw through a glass surface, and withdrawal latency was recorded^56^. In brief, mice were placed in plexiglass cubicles on a glass surface. The thermal nociceptive stimulus originated from a projection bulb below the glass surface, and the stimulus was delivered separately to one hind paw at a time. Latency was defined as the time required for the paw to show a brisk withdrawal. Each hind paw was tested three times with intervals, and the average withdrawal latency was calculated.

Nocifensive response (shaking) was measured as a quantitative scale of pain responses to a 2.0-g von Frey filament applied to both hind paws. Cold allodynia was assessed by calculating nocifensive responses for 45 s after application of one drop of acetone each to both hind paws. Mechanical hyperalgesia (pinprick) was tested with a safety pin (23-G needle, BD), and nocifensive responses were recorded. The average number of shakes of the two hind paws was used.

### Inverted screen test

To assess limb function, an inverted screen test was used^19^. Mice were placed in the center of the wire screen (width 7 mm and diameter 2 mm for wire; GMC500) and rotated to an inverted position over 2 s, with the mouse’s head declining first. The screen was positioned steadily 45–50 cm above a soft material padded surface. The time was recorded until the mouse fell off (hanging time); animals were removed from the screen when they reached the cutoff point (8 min).

### Clip squeeze test

To check joint tenderness, after 1 h of incubation in a Hargreaves’ box (IITC), a toothless clip (420 G) was applied to squeeze the proximal interphalangeal joint and extension of the metatarsal–phalangeal joint of one hind paw for 5 s. Shaking numbers were then analyzed for 4 min after clip removal.

### Sunflower seed test

To test forepaw dexterity, the sunflower seed test was used (modified from previous descriptions^57,58^). Animals were habituated to separate test boxes (animal enclosure, IITC) placed on a gray matte acrylic floor. After habituation, two to three sunflower seeds, provided by KM-B, KI (Komparativ Medicin Biomedicum, Karolinska Institutet), were left on the floor for 20 min for three consecutive days (one round of training). Only activated mice (those that completely deshelled the seeds, around 60% of C57BL/6N mice and 100% of BALB/c mice) after two rounds of training were used for further study. Two days before testing (habituation days 1 and 2) and during the testing day (testing on day 3), animals were transferred from their home cages, placed in the test box and allowed to explore the environment for 10 min. Two seeds were thereafter placed on the floor, and seed eating activity was recorded for 20 min. Episodes of rotation were calculated (the act of manipulating shell orientation by rotating 180° within the forepaws).

### Gain-of-function study

Light threshold was defined as the lowest light intensity provoking a withdrawal (reflex) or nocifensive response (shaking) in one of the hind paws. Subthreshold light stimulations were then applied simultaneously with sensory stimuli. The subthreshold light intensities for baseline mice and mice with arthritis were adjusted for hyperexcitability associated with arthritis (*Wnt1*-ChR2 mice baseline reflex subthreshold: 12.7 × 10^−3^ mW mm^−2^, nocifensive subthreshold: 18.5 × 10^−3^ mW mm^−2^; mice with arthritis reflex subthreshold: 11.7 × 10^−3 ^mW mm^−2^, nocifensive subthreshold: 14.5 × 10^−3^ mW mm^−2^; *Mrgprd*^ChR2^ mice no difference in subthreshold light; *TrkA*^ChR2^ mice baseline reflex subthreshold: 19.7 × 10^−3^ mW mm^−2^, mice with arthritis reflex subthreshold: 15.4 × 10^−3^ mW mm^−2^). No effects were found in *Sst*^ChR2^ or *Vglut3*^ChR2^ mice with the subthreshold gain-of-function blue light in normal mice or mice with arthritis.

### Inhibitory optogenetics

Animals were tested before and after yellow light. A yellow LED plate (wavelength: 566 nm; 0.1 mW mm^−2^) was positioned under the testing floor with exposures of 30 min for *TrkA*^ArchT^ mice and 45 min for *Gfra3*^ArchT^ mice (*n* = 8). For mechanical sensitivity, after 1 h of habituation on a mesh floor, the plantar surface of the hind paws was stimulated with a series of calibrated monofilaments (Stoelting) with increasing force (0.07, 0.16, 0.4, 0.6, 1.0, 1.4 and 2.0 g). Each filament was applied five times to both hind paws. The percentage of animals with a withdrawal reaction was reported.

### Joint innervation and function

Retrograde tracing of sensory neurons was performed by intra-articular ankle joint injection of 5 µl of AAV-retro-CAG-eGFP virus (2 × 10^12^ viral genomes per ml, v24-retro, ETH Zurich Virus Core) administered to C57BL/6N mice. Functional activation was achieved by injection of AAV-retro-hSyn1-hChR2(H134R) mCherry virus (5.3 × 10^12^ viral genomes per ml, v124-retro). A blue laser (470 nm, 10 Hz, 50 ms ON/OFF) was applied to the ankle joints for 2 min, and light threshold was determined as the lowest light intensity provoking a withdrawal (reflex) or nocifensive response before (baseline) and after arthritis was established (days 3, 12 and 31).

### DRG single-cell suspension preparation

Cervical and lumbar DRGs from C57BL/6N mice (10–15 weeks, control and arthritis) at different timepoints (0.25, 0.5, 1, 2, 12, 33 and 63 days) were dissected in a 6-cm Petri dish with DPBS (Sigma) on ice. Single-cell suspensions were generated largely according to our previous protocol with modifications^59^. In brief, dissected DRGs were chopped with microscissors one to two times in 2 ml of papain (25 U ml^−1^; Worthington Biochemical) and incubated for 30 min at 37 °C with a mixture of digestion enzymes including papain/collagenase/dispase (papain, 25 U ml^−1^, 4 ml; DNase I, 55 U ml^−1^, 0.5 ml, Worthington Biochemical; collagenase and dispase 20 mg ml^−1^, 800 μl, Worthington Biochemical). DRGs were triturated up and down ten times using glass Pasteur pipettes with decreasing diameter (precoated with 0.5% bovine serum albumin (BSA)). Cell suspensions were filtered through a 30-µm cell strainer (CellTrics, Sysmex) and washed with an additional 1.5 ml of artificial cerebrospinal fluid (ACSF; 87 mM NaCl, 2.5 mM KCl, 1.25 mM NaH_2_PO_4_, 26 mM NaHCO_3_, 75 mM sucrose, 20 mM glucose, 0.5 mM CaCl_2_ and 4 mM MgSO_4_) and 0.5 ml of DPBS. Cells were pelleted by centrifugation (300*g* × 6 min, 4 °C) and resuspended with 1.5 ml of cold ACSF with 0.5 ml of DPBS. The cell suspension was carefully loaded on top of the same volume of OptiPrep density gradient medium (Sigma) and centrifuged at 700*g* for 10 min at 4 °C. The cell pellet was resuspended with 3 ml of cold ACSF. SYTOX Blue (Invitrogen, Thermo Fisher Scientific) was added to stain dead cells. Thereafter, live SYTOX Blue-negative cells were sorted by fluorescence activated cell sorting (BD FACSAria Fusion/BD FACSAria III) at 4 °C. Cells were concentrated by centrifugation (300*g* × 5 min, 4 °C) and resuspended in a proper volume (~1,000 cells per µl) of ACSF solution.

### Single-cell gene expression 3′ sequencing

Sorted cells were loaded onto a 10x Chromium Chip G to yield single-cell droplets with a v3.1 kit (10x Genomics). Targeted cell recovery for each well was set to 5,000 cells (∼10% neurons). Reverse transcription, cDNA amplification and library construction were performed according to the user guide provided by 10x Genomics, and libraries were sequenced at the National Genomics Infrastructure (SciLifeLab) and aligned to mouse reference mm10 using the STAR aligner to generate the gene–cell matrices.

### scRNA-seq data analysis

The R (v.4.1.1) package Seurat (v.4.1.0) was used for the main scRNA-seq analysis. Individual count matrices created by CellRanger (v.5.0.1) were merged to a single Seurat object, and all cells with more than 20% of counts originating from mitochondrial genes were discarded. A cutoff at more than 2,000 detected genes was set for the primary data. These data were integrated using Harmony (v.0.1.0) and clustered using the default algorithm in Seurat. Putative neuronal clusters were identified using the neuronal marker gene *Rbfox3*. Non-neuronal clusters from control samples were extracted, integrated, clustered and assigned cell labels according to Krauter et al.^10^. The non-neuronal control data were then used to transfer labels (Seurat) to all remaining non-neuronal data. After this, all remaining original data with ≥1,000 detected genes were integrated, clustered and assigned labels from the primary data. All neurons from these secondary data were discarded to make sure that only high-quality neurons were used for the final analyses. Then, the primary (>2,000 detected genes) and secondary (≥1,000 detected genes) datasets were merged to produce the full working dataset. More granular identities for the immune cells in the data were assigned using a peripheral nerve immune cell atlas^60^. For this, a mixture discriminant analysis (mda)-based classifier (scPred, v.1.9.2) was built using these data, and the cell-type labels for the immune cells in the present data were learned using this model. All cells with a prediction score below 0.55 were discarded. For neurons, all cells labeled as neurons were extracted from the full working data and clustered, and using iterative clustering steps all cells with less than 0.5 normalized counts of *Rbfox3* and more than two normalized counts of *Apoe* were removed. A classifier was then built as described before using data from Zeisel et al.^13^ with annotations from Usoskin et al.^12^, cell-type labels for the neuronal data were learned using the model, and unassigned neurons were discarded similar to as stated above. For a pseudobulk differential expression analysis, neuron types were collapsed together, and data from each individual time point after inducing arthritis were compared against controls (*t*_0_) using a Wilcoxon rank-sum test with the Seurat function FindMarkers and an adjusted *P* value cutoff of 1 × 10^−20^. Differentially expressed genes for each cell type between individual arthritis timepoints and controls were defined in a similar fashion. Fcoex (v.1.10.0) was used to identify co-regulated gene modules in the dataset. For this, to reduce computational load, a random set (25%) of cells from each cell type–timepoint pair was sampled. Fcoex was run for the first 200 genes using ‘timepoint’ as the target. The resulting set of modules was further filtered to contain only differentially expressed genes and modules that consisted of a minimum of ten genes. A module score was calculated for modules and scaled to fall between 0 and 1. A GSEA for gene modules was run using enrichR (v.3.0) with the ‘GO_Biological_Process_2021’ and ‘KEGG_2019_Mouse’ databases. For the perturbation analysis (Augur v.1.0.0), all genes situated on the Y chromosome and non-protein-coding genes were first discarded. Following this, the analysis was run comparing each individual timepoint against controls for each neuron type. The default minimum of 20 cells per type/timepoint was used; therefore, some neuron types were not compared for each timepoint. For hallmark GSEA, we used MSigDB^61^. SCENIC was used to infer master transcription factor regulons across all cell types and timepoints^62^.

### Intercellular ligand–receptor analysis

Upregulated genes at the 12-h timepoint were compared to the 0-h timepoint by the following criteria: greater than threefold change, expression in >10% of cells within a cell type and *P* < 0.05. Ligand-encoding genes were selected, and all expressed genes were used for determining the receptor genes in each cell type. Ligand–receptor pairs were calculated using the ligand–receptor dataset^63^. We then collected associated gene patterns for each ligand–receptor pair from NicheNet and SCENIC^62,64^. Ligand–receptor activity was scored using the enrichment score^65^. In brief, we used all upregulated genes as the candidate gene pool. Ligand–receptor-associated gene patterns were selected for scoring, whereas the remaining genes were considered background genes and ranked based on their expression. We created 20 intervals according to background gene expression and randomly selected 100 genes from each interval to form a random background gene matrix. Feature scores were calculated by comparing the average expression of the associated genes to the randomized background genes. All negative values were normalized to 0, indicating nonactivity. To reduce variation, for each ligand–receptor pair between two cell types, the enrichment score was calculated five times with the average value as the activity score. The specific ligand–receptor pairs between different cell types were visualized in a Sankey plot.

### Type 1 IFN signaling blockade

C57BL/6N mice received either a neutralizing monoclonal antibody to IFNAR1 (40 mg per kg (body weight), i.p., BioXCell) or an isotype of mouse IgG1 (40 mg per kg (body weight), i.p., BioXCell) 1 h before the injection of cartilage autoantibodies (day 0) or on day 22 or day 45 after autoantibody injection (*n* = 6). TYK2 inhibitor (deucravacitinib/MBS-986165, MCE, in ethanol:TPGS:PEG300 (5:5:90)) was orally administrated twice daily for 9 days from day 13 after autoantibody injection in C57BL/6N mice (8:00 AM and 8:00 PM, 15 mg per kg (body weight); *n* = 6)^66^. A single i.p. injection of MNK1/MNK2 inhibitor, tomivosertib (eFT508/HY-100022, MCE, 1 mg per kg (body weight), in DMSO:PEG300:Tween-80:saline (5:40:5:50)), was administered on day 48 after antibody injection in C57BL/6N mice (*n* = 10, 5 females and 5 males). eIF4E/eIF4G interaction inhibitor, 4EGI-1 (324517, Sigma), was i.p. injected into C57BL/6N mice with antibody-induced arthritis (15 mg per kg (body weight) in DMSO:PEG300:Tween-80:saline (5:40:5:50); *n* = 5) on day 56. Animals were tested in behavioral tests as described in the Results.

### *Ifnar1* knockout in the DRG

Knockout of *Ifnar1* was performed with AAV-PHP.S-CAG-eGFP_Cre virus (1.7 × 10^13^ viral genomes per ml, v25-PHP.S, ETH Zurich Virus Core) in *Ifnar1*^*fl/fl*^ mice. AAV-PHP.S-CAG-eGFP virus (1.3 × 10^13^ viral genomes per ml, v24-PHP.S, ETH Zurich Virus Core) served as the control virus. Somatic CRISPR knockout was performed using a hybrid strategy combining constitutive Cas9 expression with AAV-mediated delivery of gRNAs, AAV-PHP.S-U6>*Ifnar1*(gRNA 1)-H1>*Ifnar1*(gRNA 4)-7SK>*Ifnar1*(gRNA 5) virus (3.58 × 10^13^ viral genomes per ml, AMSBio) in Rosa26-Cas9 mice. The AAV construct contained three gRNA sequences (5’-TTCAGCAGAATATCGAACGT-3’, 5’-AAGGGAACAGCACATCTTCG-3’ and 5’-CGGACAAGACGGGAACATGT-3’) targeting the second, third and fourth exons of *Ifnar1*, respectively. AAV-PHP.S-CAG-eGFP_Cre (1.7 × 10^13^ viral genomes per ml, v25-PHP.S, ETH Zurich Virus Core, for CRISPR–Cas9) was used as the control virus. The virus (8 µl) was delivered through intrathecal injection. Mice were used for antibody-induced arthritis experiments after 6–8 weeks of virus injection. Knockout efficiency was evaluated by RNAscope experiments for *Ifnar1* expression.

### EndoS treatment of autoantibody

For Fc N-glycan cleavage, glutathione *S*-transferase-fused EndoS expressed by *Escherichia coli* was used to incubate with the cartilage antibody cocktail at a ratio of 1:1,000 (wt/wt) and 37 °C for 1 h. All antibodies were purified by using Protein G GraviTrap columns (Cytiva) according to the manufacturer’s instructions.

### Cytokine measurements in serum

Collected blood samples were centrifuged at 1,000*g* for 10 min at 4 °C, and serum samples were aliquoted and stored at –80 °C until use. Serum levels of IFNα and IFNβ as well as 31 other cytokines/chemokines were measured by a multiplex assay service (Eve Technologies) using a Mouse IFN 2-Plex Discovery Assay (MDIFNAB) and Mouse Cytokine/Chemokine 31-Plex Discovery Assay Array (MD-31), respectively (*n* = 4).

### qPCR of IFNs in DRG samples

Fresh DRG samples were collected at different timepoints after autoantibody injection: 1 h, 12 h, 3 days, 33 days and 63 days, four mice for each group, whereas the 12 h group had eight mice. Naive C57BL/6N mice (*n* = 9) were used as the control group. For mice treated with anti-IFNAR1, samples were collected 33 days after autoantibody injection with 12 h of treatment with monoclonal anti-IFNAR1 (40 mg per kg (body weight), i.p., BioXCell; *n* = 5). Fresh-frozen human lumbar DRGs were used and are listed in Supplementary Table 8. Total RNA was extracted from mouse cervical and lumbar DRGs using TRIzol Reagent (Thermo Fisher Scientific) and a Motorized Pestle Mixer (Argos Technologies), as previously described^67^. cDNA was generated from 500 ng of RNA using a High-Capacity cDNA Reverse Transcription kit (Applied Biosystems) with random primers according to the manufacturer’s instructions. qPCR reactions were performed using SYBR Green Master Mix (Thermo Fisher Scientific) on a QuantStudio5 System (Applied Biosystems). Primer pairs used in this study are listed in Supplementary Table 9. All assays were performed in duplicate, and the levels of transcripts were analyzed by the comparative cycling threshold (${2}^{-\mathrm{\varDelta \varDelta }{C}_{{\mathrm{t}}}}$) method relative to mouse *Gapdh* or human *GAPDH*.

### Tissue preparation and in situ hybridization (RNAscope)

Mice were deeply anesthetized and decapitated. Lumbar DRGs were dissected, snap-frozen and maintained at −80 °C. Fresh-frozen human lumbar DRGs were provided by AnaBios (L3–L5 DRGs; Supplementary Table 8). Twelve-micron sections were mounted onto Superfrost Plus microscope slides and stored at −80 °C until use.

RNAscope assays were performed according to the protocol provided with an RNAscope Multiplex Fluorescent Detection kit v2 (323110, ACDBio) with minor modifications, where the hydrogen peroxide treatment was omitted, protease 3 was used instead of protease 4, and counterstaining with DAPI (1.0 μg ml^−1^, 10 min at room temperature) was performed. Slides were mounted after rinsing in PBS with mounting medium (Agilent Dako), dried overnight at room temperature and stored at −20 °C until imaging. The probes included in this study were designed and provided by ACDBio as listed in Supplementary Table 9.

### Immunohistochemistry

Mice were deeply anesthetized with sodium pentobarbital (300 mg per kg (body weight)) and perfused transcardially with 20 ml of prewarmed (37 °C) saline, followed by 20 ml of prewarmed 4% paraformaldehyde in 0.16 M phosphate buffer (pH 7.2–7.4) and 50 ml of cold fixative. L4/L5 DRGs were dissected and postfixed in the same fixative for 90 min at 4 °C. After cryoprotection in 10% sucrose with 0.1 M phosphate buffer containing 0.01% sodium azide (VWR) and 0.02% bacitracin (Sigma) for 48 h, the tissue was embedded with OCT (HistoLab), frozen with liquid carbon dioxide and sectioned on a CryoStar NX70 cryostat (Thermo Scientific) at 12-µm thickness. Immunohistochemistry was conducted as described in Zhang et al.^68^ using antibodies listed in Supplementary Table 9.

### H&E staining

Ankle joints were collected from BALB/c mice on day 60 after autoantibody injection after transcardial perfusion and then postfixed in 4% paraformaldehyde overnight at 4 °C. After decalcification in EDTA solution (Sigma), the joints were cryosectioned and stained with H&E (Sigma). Histopathological analysis was evaluated by a three-grade system for synovitis (inflammation), cartilage destruction and bone erosion as previously described^54^, five sections for each animal.

### Microscopy, image processing and quantitative analysis

Representative confocal images were acquired from 1-Airy unit pinhole on an LSM700/LSM800/LSM900-Airy confocal laser-scanning microscope (Carl Zeiss) and limited emission spectra for image acquirement. Joint histology images were acquired by microscopy using an Olympus IX73. For mRNA or protein intensity analysis, cell segmentation was performed with cellpose, and intensity was detected with ImageJ. Images were processed using ZEN2012 software (Zeiss).

### Patch clamp electrophysiology

Cell cultures for patch clamp electrophysiology were prepared from adult C57BL/6N mice (both sexes, 7–10 weeks). In brief, all levels of cervical and lumbar DRGs were dissected and digested in a mixture of papain/collagenase/dispase and triturated using glass Pasteur pipettes. Dissociated cells were suspended in L-15 medium (Liebovitz, L1518, Merck) containing 10% fetal bovine serum, NaHCO_3_, glucose, penicillin/streptomycin (1×) and floxuridine (PHR2589, Merck) and plated on coverslips precoated with poly-d-lysine (A-003-E, Merck) and laminin (L2020, Merck). The following day, changes in neuronal excitability in small-sized nociceptors (diameter of ≤20 µm) were tested after a 1-h preincubation in recombinant mouse IFNα protein (12100-1, 300 U ml^−1^, B&D Systems); PBS containing 0.1% BSA (Sigma) stimulation in neurons served as the control. To test the effects of MNK1/MNK2 inhibition on IFN-stimulated nociceptors, neurons were first incubated with 10 µM eFT508 (in L-15 medium) or vehicle (DMSO, 0.1% (vol/vol)) for 1 h, and IFNα (300 U ml^−1^) was then added. After 1 h of IFNα incubation, the coverslip was placed in a 35-mm Petri dish filled with ACSF solution. ACSF was composed of 125 mM NaCl, 25 mM glucose, 25 mM NaHCO_3_, 2.5 mM KCl, 2 mM CaCl_2_, 1.25 mM NaH_2_PO_4_ and 1 mM MgCl_2_ that was saturated with 95% oxygen and 5% carbon dioxide and maintained at room temperature (20–22 °C).

All recordings were performed using a whole-cell patch clamp technique. Patch clamp electrodes were filled with a solution containing 120 mM K-gluconate, 5 mM KCl, 10 mM HEPES, 4 mM Mg_2_ATP, 0.3 mM Na_4_GTP and 10 mM sodium phosphocreatine with pH 7.4 adjusted with KOH and an osmolarity of 275 mOsm. Neurons were visualized using a fluorescence microscope (Axioskop FS Plus, Zeiss) equipped with IR-differential interference contrast optics and a CCD camera (Hamamatsu). Patch clamp electrodes were advanced into the dish using a motorized micromanipulator (Luigs and Neumann) while applying constant positive pressure. Intracellular signals were amplified using a MultiClamp 700B amplifier (Molecular Devices) and low-pass filtered at 10 kHz. Electrophysiological data were digitized at 10 or 20 kHz using a Digidata 1322AA/D converter (Molecular Devices) and acquired using pClamp software (Molecular Devices). Neurons were held at –60 mV in current clamp mode, and ramps of depolarizing current (1-s duration, peak amplitudes of 100, 300, 500 and 700 pA) were injected into each neuron. The total number of action potentials elicited by each current ramp was quantified for each neuron.

### Western blotting

To perform western blotting, cervical and lumbar DRGs were collected at different timepoints after antibody injection: 1 h, 12 h and 33 days, four mice for each group. For mice treated with anti-IFNAR1, samples were collected at 33 days after 12 h of treatment with monoclonal anti-IFNAR1 (40 mg per kg (body weight), i.p., BioXCell; *n* = 3). Naive C57BL/6N mice (*n* = 9) were used as the control group. Total protein was extracted using N-PER neuronal protein extraction reagent (87792, Thermo Fisher Scientific) containing protease inhibitor cocktail (G6251, Promega) and Halt phosphatase inhibitor cocktail (78428, Thermo Fisher Scientific). Tissues were homogenized using a micropestle (Sigma), followed by sonication, incubation for 10 min on ice, centrifugation at 10,000*g* and collection of supernatants. Total protein concentration was measured with a BCA assay kit (Thermo Fisher Scientific). Protein lysates from fresh human lumbar DRGs from healthy donors, individuals with RA with pain and individuals with RA without pain (AnaBios, Supplementary Table 8) were homogenized with beads using a tissue homogenizer (Qiagen) and processed in a similar way to mouse DRGs. For mouse DRG lysates, 20 µg of denatured protein was separated by electrophoresis on a NuPAGE 4–12% Bis-Tris gel and transferred onto a 0.2-µm nitrocellulose membrane with an iBlot2 transfer system. Membranes were blocked with 5% BSA in 0.1% Tween-20 (TBST) for 1 h at room temperature and then incubated with primary antibody to p-eIF4E (Ser209, Cell Signaling Technology) for 2 days at 4 °C. Membranes were washed in TBST three times for 15 min each at room temperature on a shaker and incubated with polyclonal secondary antibody conjugated with horseradish peroxidase (HRP) in 5% BSA (Dako) for 1 h at room temperature. Signal was detected with SuperSignal West Femto reagents (Thermo Fisher Scientific) after washing the membrane and imaged with a ChemiDoc MP system (Bio-Rad Laboratories). Membranes were stripped in Restore Plus Western Blot stripping buffer (46430, Thermo Fisher Scientific) for 1 h, washed with TBST (three times, 15 min each), blocked with 5% BSA and reprobed with primary antibody to eIF4E (Cell Signaling Technology) overnight at 4 °C. The membrane was washed, incubated with HRP-conjugated secondary antibody, detected with Amersham ECL Prime Western Blotting detection reagents and imaged with a ChemiDoc system. At the end, GAPDH (Cell Signaling Technology) was probed on the stripped membranes as the loading control. Band intensity were quantified with Image Lab 6.1 software (Bio-Rad Laboratories). Protein phosphorylation levels were normalized to total protein expression and compared to normalized control samples. For human DRG lysates, 30 µg of denatured protein was loaded for western blotting and probed with primary antibodies (IFNα from Thermo Fisher Scientific and β-actin from Abcam) in a similar protocol as described above using antibodies listed in Supplementary Table 9.

### Ex vivo teased tibial nerve recordings

Extracellular recordings from single cutaneous primary afferent axons in an isolated mouse glabrous skin–tibial nerve preparation were obtained following previously published procedures^69,70^. Three months (days 85–98) after injection with either autoantibody (arthritis) or saline (control; *n* = 5, both males and females), mice were killed by cervical dislocation, and the glabrous skin from one hind paw with the tibial nerve attached was dissected and placed in a custom-made two-compartment Teflon recording chamber with the corium side down. The chamber containing the preparation was continuously superfused at a rate of 5 ml per min with oxygenated external solution consisting of 107.8 mM NaCl, 26.2 mM NaHCO_3_, 9.64 mM sodium gluconate, 7.6 mM sucrose, 5.55 mM glucose, 3.5 mM KCl, 1.67 mM NaH_2_PO_4_, 1.53 mM CaCl_2_ and 0.69 mM MgSO_4_, which was adjusted to pH 7.4 by continuously gassing with 95% O_2_, 5% CO_2_. The temperature was maintained to ±1 °C using a heat exchanger connected to a thermostat. The tibial nerve was placed into an adjacent chamber of the bath filled with mineral oil and then teased into small bundles that were individually placed on a gold wire electrode. A reference electrode was positioned inside the recording chamber dipped into the aqueous solution. Input signals were amplified through a high-gain AC differential amplifier (Neurolog NL104A, Digitimer), digitized (PowerLab 8, ADInstruments) at 25 kHz and stored in the hard drive of a PC for offline analysis. The LabChart software package (ADInstruments) was used for recording and offline analysis. Mechanically responsive receptive fields were identified by probing the skin flap with a blunt glass rod. Once a suitable fiber was found, a mechanical stimulator consisting of a tension/length feedback controller (300C-I, Aurora Scientific) was used to apply mechanical stimuli. Two different force protocols were used to characterize mechanical responses. Threshold and firing frequencies were measured during continuous force ramps from 0 to 100 mN (ramp duration 10 s) and firing frequencies during static force applications (0 to 5, 10, 20, 40, 50, 75, 150 and 200 mN, step duration 10 s; 50-s interforce interval). Only mechanically responsive C-fibers (conduction velocity of <1.2 m s^−1^) were used in these experiments^71^. The experimenter was blinded to genotype until data analysis was complete.

### Statistics and reproducibility

Behavior data for the von Frey filament up–down test and nocifensive behavioral tests (including the 2-g von Frey, acetone cold allodynia, pinprick and squeeze tests) as well as the sunflower seed assay (noncontinuous data) are presented as median with interquartile range. Heat hypersensitivity, inverted screen test data, western blotting quantification data and cytokine levels in sera are presented as mean ± s.d. Clinical scores and qPCR data are shown as mean ± s.e.m. A *P* value less than 0.05 was considered significant. Data were analyzed with Prism 10.2.0 (GraphPad software) as specified in the figure legends. The sample size was determined according to our previous experience and publication^54^. Animals for the experimental groups were randomly assigned. Animal exclusion was applied to those that were not suitable for behavioral tests during the testing day. Animal behavioral assessments were blinded during testing and quantification of videos. Skin nerve recordings and patch clamp recordings were blinded. Single-cell data analysis was not blinded.

### Reporting summary

Further information on research design is available in the Nature Portfolio Reporting Summary linked to this article.

## Online content

Any methods, additional references, Nature Portfolio reporting summaries, source data, extended data, supplementary information, acknowledgements, peer review information; details of author contributions and competing interests; and statements of data and code availability are available at 10.1038/s41593-026-02234-y.

## Supplementary information

Supplementary Information Supplementary Figs. 1–4.

Reporting Summary

Supplementary Table 1 Supplementary Tables 1–9.

Supplementary Video 1 Sunflower seed assay in a control mouse (C57BL/6N male).

Supplementary Video 2 Sunflower seed assay in a mouse with arthritis (day 33 after antibody injection).

## Source data

Source Data Fig. 1 Statistical source data.

Source Data Fig. 2 Statistical source data.

Source Data Fig. 3 Statistical source data.

Source Data Fig. 5 Statistical source data.

Source Data Fig. 6 Statistical source data.

Source Data Fig. 7 Statistical source data.

Source Data Extended Data Fig. 1 Statistical source data.

Source Data Extended Data Fig. 2 Statistical source data.

Source Data Extended Data Fig. 3 Statistical source data.

Source Data Extended Data Fig. 7 Statistical source data.

Source Data Extended Data Fig. 8 Statistical source data.

Source Data Extended Data Fig. 9 Statistical source data.

Source Data Extended Data Fig. 10 Statistical source data.

## Data Availability

The RNA-seq datasets generated and analyzed during the study have been deposited in the Gene Expression Omnibus (GEO) repository under a SuperSeries accession number GSE218634. Source data are provided with this paper.

## Code availability

The code and analysis steps for the intercellular ligand–receptor analysis are accessible as a Jupyter Notebook on https://sccamel.readthedocs.io/. Previously published code and software used for this study are cited and described in the Methods.
