# Supplementary material for: Persistent interferon signaling causes sensory neuron plasticity and pain before and during arthritis
Source: Nat Neurosci. 2026 Mar 10;29(5):1095–108. doi: 10.1038/s41593-026-02234-y (PMC13156039; doi:10.1038/s41593-026-02234-y)
Supplement: Supplementary file 1 — Supplementary Figs. 1–4. [file 41593_2026_2234_MOESM1_ESM.pdf]

nature neuroscience

Supplementary information

https://doi.org/10.1038/s41593-026-02234-y

Persistent interferon signaling causes sensory neuron plasticity and pain before and during arthritis

In the format provided by the authors and unedited

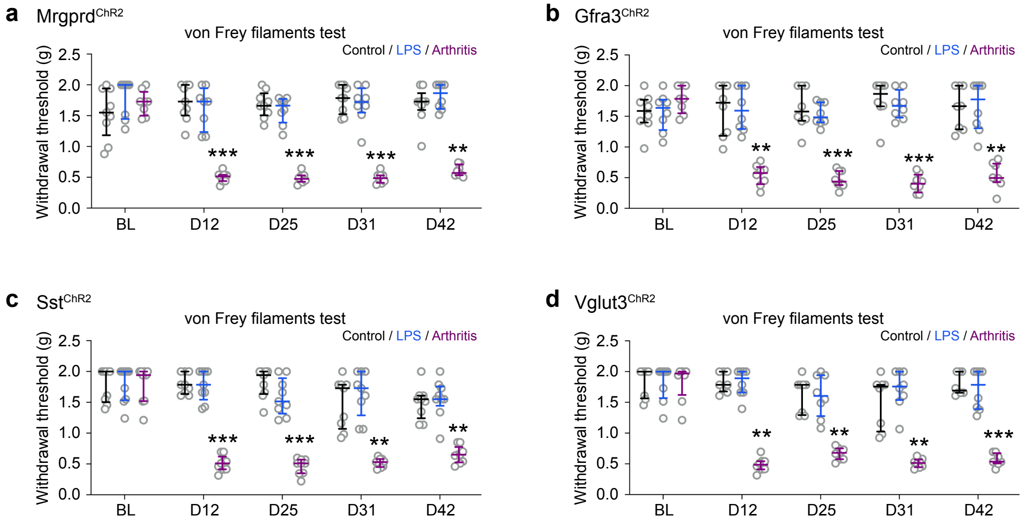

Supplementary Fig. 1 Mechanical sensitivity in different mouse strains in control, LPS and mice with LPS and autoantibody (Arthritis). Withdrawal threshold was measured before (BL) and at the indicated timepoints (n = 8/9 mice/group for each mouse strain). von Frey filament tests data in (**a**-**d**) were analyzed with Kruskal-Wallis test followed by Dunn's multiple comparisons test at each time point. Calculated *P* values for control vs LPS and control vs Arthritis are listed below. BL: 0.3499, >0.9999, D12: >0.9999, 0.0007, D25: >0.9999, 0.0006, D31: >0.9999, 0.0007, D42: >0.9999, 0.0028 in (**a**); BL: 0.9999, >0.3638, D12: >0.9999, 0.0023, D25: >0.9999, 0.0007, D31: >0.9999, 0.0005, D42: >0.9999, 0.0021 in (**b**); BL: >0.9999, >0.9999, D12: >0.9999, 0.0008, D25: 0.8866, <0.0001, D31: >0.9999, 0.0022, D42: >0.9999, 0.0021 in (**c**); BL: >0.9999, >0.9999, D12: >0.9999, 0.0020, D25: >0.9999, 0.0016, D31: >0.9999, 0.0050, D42: >0.9999, 0.0008 in (**d**). ** indicates $P < 0.01$, *** indicates $P < 0.001$.

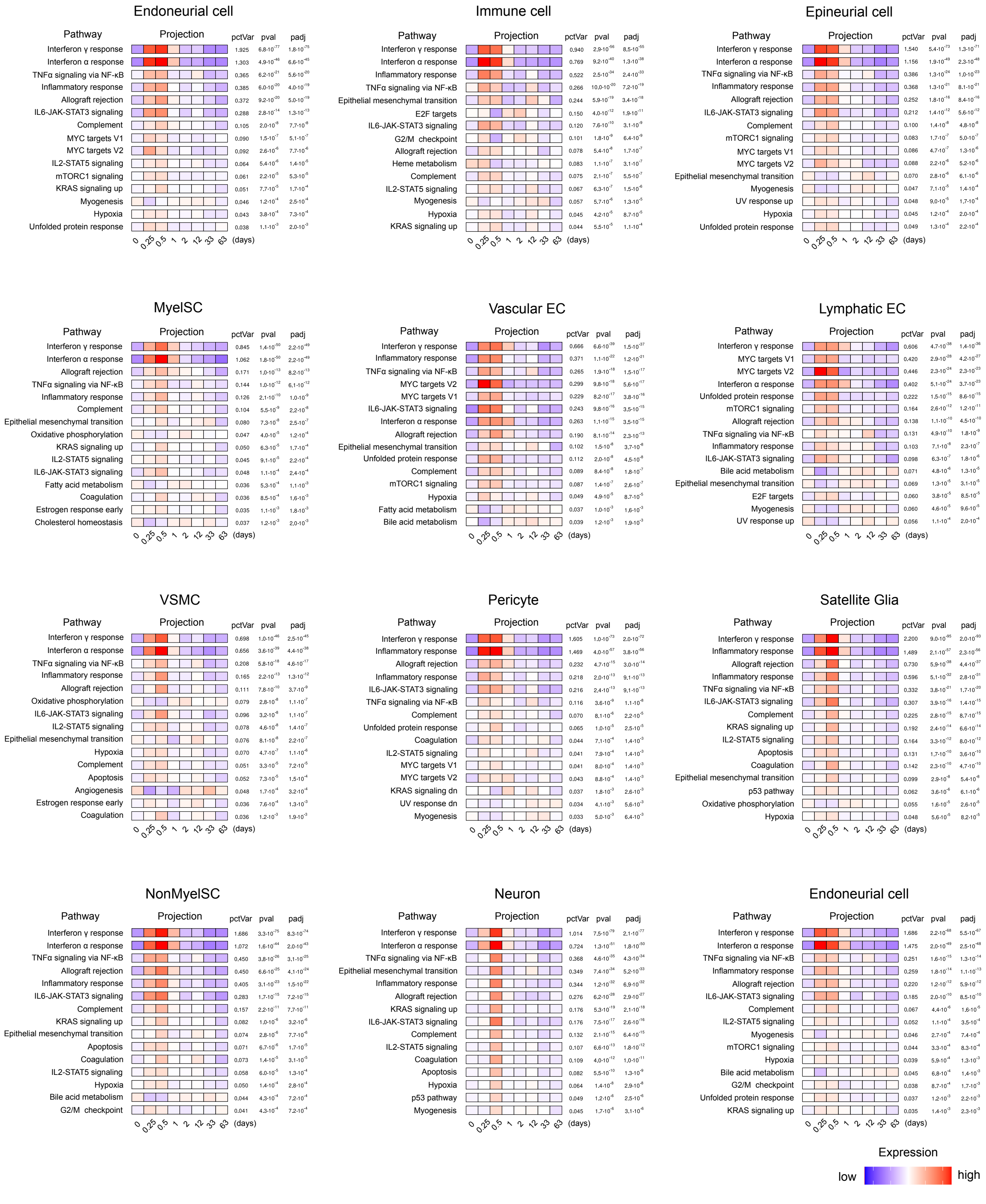

Supplementary Fig. 2 Gene-set enrichment analysis (GSEA) of DRG cell types. GSEA analysis for hallmark genes in DRG cell types at different timepoints after injection of cartilage autoantibodies in mice.

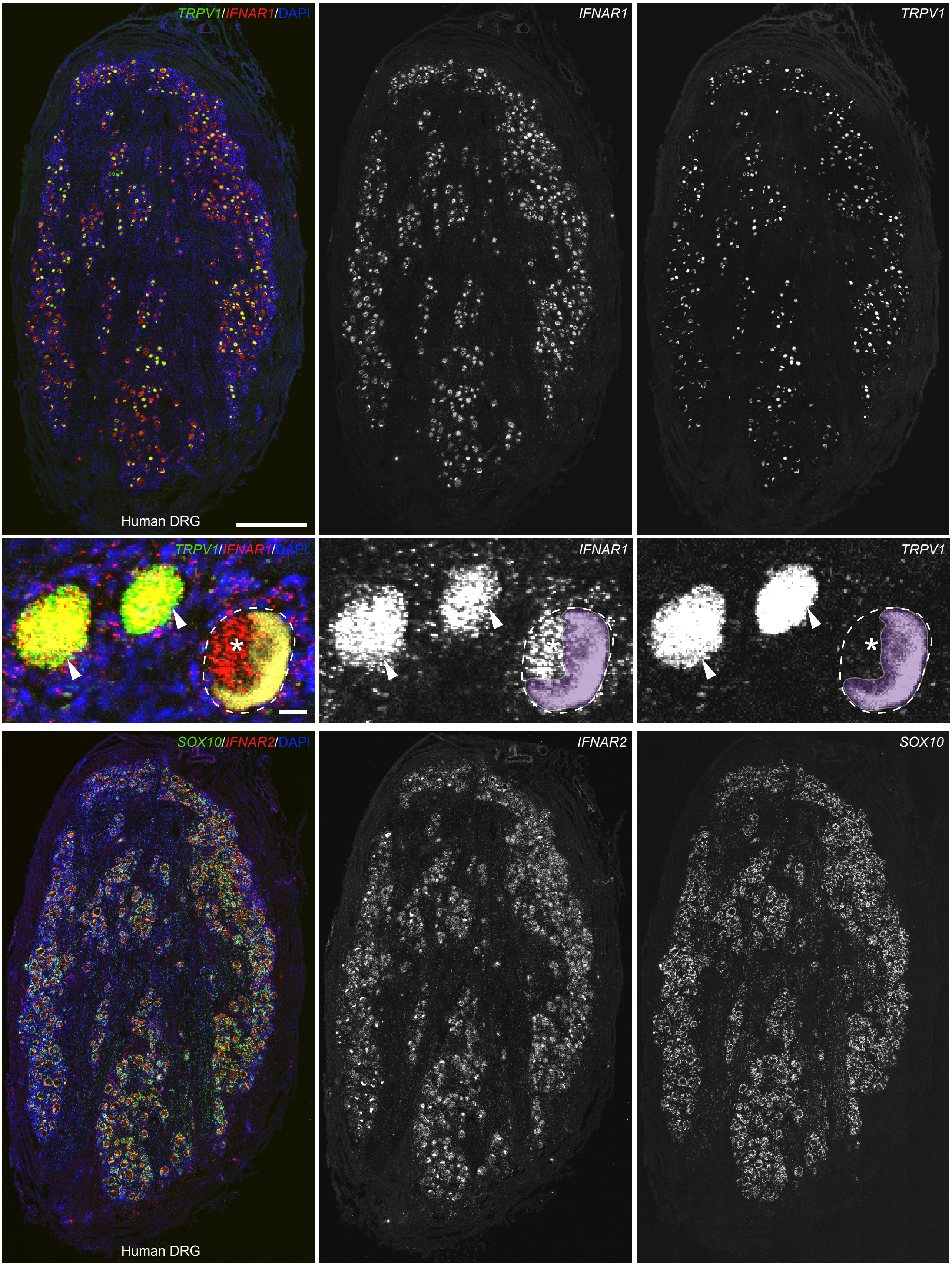
TRPV1/IFNAR1/DAPI
IFNAR1
TRPV1
Human DRG
TRPV1/IFNAR1/DAPI
IFNAR1
TRPV1
SOX10/IFNAR2/DAPI
IFNAR2
SOX10
Human DRG

Supplementary Fig. 3 Expression of *IFNAR1* and *IFNAR2* mRNAs in human DRGs detected by RNAscope. Top: Representative overview image of co-expression of *TRPV1* and *IFNAR1* mRNAs in human DRG counter stained with DAPI. Middle: High magnification image of co-localization between *TRPV1* and *IFNAR1* mRNAs in DRG neurons as indicated by arrowheads. The asterisk indicates autofluorescence caused by accumulated lipofuscin. Bottom: Representative overview image of *SOX10* and *IFNAR2* mRNA expression in human DRG counter stained with DAPI. Scale bars: 1 mm for overview images on top and bottom panels and 20 µm for middle panel.

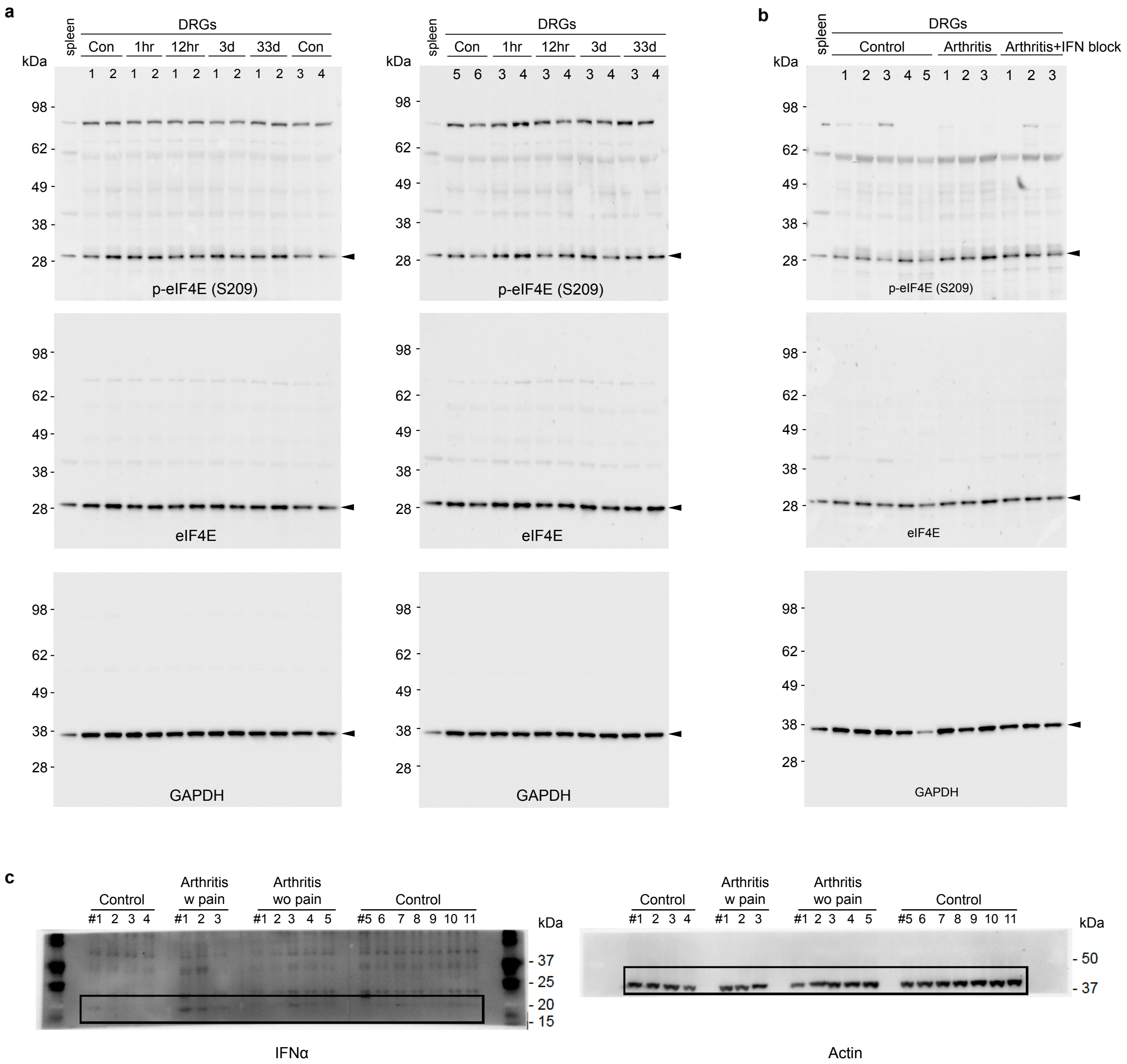

Supplementary Fig. 4 Western blotting of phospho-eIF4E (Ser209) in DRGs of cartilage autoantibody injected mice, and IFNα in human DRGs. (**a**) Western blotting of phospho-eIF4E (Ser209), eIF4E and GAPDH in DRG lysates from control and cartilage autoantibody injected mice at different timepoints. (**b**) Western blotting of phospho-eIF4E (Ser209), eIF4E and GAPDH in DRG lysates from control, arthritis mice (d33) and arthritis mice treated with IFNAR1 antibody (IFN block). Arrowhead indicates the specific band. Spleen sample was added as positive control. DRG samples from different mice are numbered at the top of the membranes. (**c**) Western blotting of IFNα and Actin in DRG lysates from healthy control, arthritis with pain and arthritis without pain patient donors. Rectangles indicate the specific bands.
