# Supplementary material for: Persistent interferon signaling causes sensory neuron plasticity and pain before and during arthritis
Source: Nat Neurosci. 2026 Mar 10;29(5):1095–108. doi: 10.1038/s41593-026-02234-y (PMC13156039; doi:10.1038/s41593-026-02234-y)
Supplement: Supplementary file 2 — Reporting Summary [file 41593_2026_2234_MOESM2_ESM.pdf]

nature portfolio

Corresponding author(s): Patrik Ernfos

Last updated by author(s): Dec 21, 2025

Reporting Summary

Nature Portfolio wishes to improve the reproducibility of the work that we publish. This form provides structure for consistency and transparency in reporting. For further information on Nature Portfolio policies, see our Editorial Policies and the Editorial Policy Checklist.

Statistics

For all statistical analyses, confirm that the following items are present in the figure legend, table legend, main text, or Methods section.

n/a	Confirmed	
☐	☒	The exact sample size (*n*) for each experimental group/condition, given as a discrete number and unit of measurement
☐	☒	A statement on whether measurements were taken from distinct samples or whether the same sample was measured repeatedly
☐	☒	The statistical test(s) used AND whether they are one- or two-sided<br>*Only common tests should be described solely by name; describe more complex techniques in the Methods section.*
☐	☒	A description of all covariates tested
☐	☒	A description of any assumptions or corrections, such as tests of normality and adjustment for multiple comparisons
☐	☒	A full description of the statistical parameters including central tendency (e.g. means) or other basic estimates (e.g. regression coefficient) AND variation (e.g. standard deviation) or associated estimates of uncertainty (e.g. confidence intervals)
☐	☒	For null hypothesis testing, the test statistic (e.g. *F*, *t*, *r*) with confidence intervals, effect sizes, degrees of freedom and *P* value noted<br>*Give P values as exact values whenever suitable.*
☒	☐	For Bayesian analysis, information on the choice of priors and Markov chain Monte Carlo settings
☒	☐	For hierarchical and complex designs, identification of the appropriate level for tests and full reporting of outcomes
☒	☐	Estimates of effect sizes (e.g. Cohen's *d*, Pearson's *r*), indicating how they were calculated

Our web collection on statistics for biologists contains articles on many of the points above.

Software and code

Policy information about availability of computer code

Data collection	Zen software (Zeiss) for confocal images, pClamp software (Molecular Devices) for patch clamp, LabChart software package (ADInstruments) for skin-neve recording.
Data analysis	R (v.4.1.1), CellRanger (v.5.0.1), Harmony (v.0.1.0), classifier (scPred, v.1.9.2), Fcoex (v.1.10.0), enrichR (v.3.0) with the “GO_Biological_Process_2021” and “KEGG_2019_Mouse” databases, pClamp software (Molecular Devices), ChemiDoc MP system (Bio-Rad Laboatories), LabChart software package (ADInstruments), Prism 10.2.0 (GraphPad software), Adobe Photoshop 2022 and Adobe Illustrator 2022 (Adobe Systems), Cellpose (3.1.0), Image J (2.0.0), BioRender.

For manuscripts utilizing custom algorithms or software that are central to the research but not yet described in published literature, software must be made available to editors and reviewers. We strongly encourage code deposition in a community repository (e.g. GitHub). See the Nature Portfolio guidelines for submitting code & software for further information.

Data

Policy information about availability of data

All manuscripts must include a data availability statement. This statement should provide the following information, where applicable:

- Accession codes, unique identifiers, or web links for publicly available datasets
- A description of any restrictions on data availability
- For clinical datasets or third party data, please ensure that the statement adheres to our policy

The RNA-seq datasets generated and analyzed during the study have been deposited in the Gene Expression Omnibus (GEO) repository under a SuperSeries accession number GSE218634 (https://www.ncbi.nlm.nih.gov/geo/query/acc.cgi?acc=GSE218634).
The codes and analysis steps of intercellular ligand-receptor analysis will be accessible as a Jupyter Notebook on https://sccamel.readthedocs.io/.

Research involving human participants, their data, or biological material

Policy information about studies with human participants or human data. See also policy information about sex, gender (identity/presentation), and sexual orientation and race, ethnicity and racism.

Reporting on sex and gender	These data are provided in Supplementary Table 8.
Reporting on race, ethnicity, or other socially relevant groupings	These data are provided in Supplementary Table 8.
Population characteristics	These data are provided in Supplementary Table 8.
Recruitment	No recruitment was required. L3-L5 human DRG were used in the study.
Ethics oversight	Approved by the Swedish Ethical Review Authority (Etikprövningsmyndigheten).

Note that full information on the approval of the study protocol must also be provided in the manuscript.

Field-specific reporting

Please select the one below that is the best fit for your research. If you are not sure, read the appropriate sections before making your selection.

☒ Life sciences ☐ Behavioural & social sciences ☐ Ecological, evolutionary & environmental sciences

For a reference copy of the document with all sections, see nature.com/documents/nr-reporting-summary-flat.pdf

Life sciences study design

All studies must disclose on these points even when the disclosure is negative.

Sample size	The sample size was determined according to our previous experience and publications.
Data exclusions	For scRNA-Seq data, cells with low quality were excluded according to the criteria of the quality control as described in the methods part of the manuscript.<br>For behavioral data, mice with signs of any unhealthy conditions or super active during the testing day were excluded according to the predefined criteria; for sunflower seed assay, animals were excluded if they did not rotate the seeds after three trainings; for excitatory optogenetics, mice were excluded if no responses to the highest intensity of the blue light in the Gfra3CreERT2-CHR2 and TrkACreERT2-CHR2 (individual variation related to recombinaion efficiency of tamoxifen)
Replication	Some in vivo experiments were performed with two replicates: behavioural experiements in C57BL/6N wildtype mice in Figure 1b-g and Extended Data Fig.1a; inhibitory optogenetics in TrkAArchT RA mice in Fig. 2c; IFNA1 mAb blocking in C57BL/6N arthritis mice in Fig.6c and Extended Data Fig. 10a; MNK inhibitor effects on RA pain in Fig. 6g and Extended Data Fig.10e. All replication attempts were successful.
Randomization	The animals for the experiments were randomized assigned.
Blinding	Animal behavioral tests were blinded during the conduct of the test and quantification of videos, Skin-nerve record and Pacth-clamp record were blinding. Single cell data analysis were not blinded.

Reporting for specific materials, systems and methods

We require information from authors about some types of materials, experimental systems and methods used in many studies. Here, indicate whether each material, system or method listed is relevant to your study. If you are not sure if a list item applies to your research, read the appropriate section before selecting a response.

Materials & experimental systems

n/a	Involved in the study
☐	☒ Antibodies
☒	☐ Eukaryotic cell lines
☒	☐ Palaeontology and archaeology
☐	☒ Animals and other organisms
☒	☐ Clinical data
☒	☐ Dual use research of concern
☒	☐ Plants

Methods

n/a	Involved in the study
☒	☐ ChIP-seq
☒	☐ Flow cytometry
☒	☐ MRI-based neuroimaging

Antibodies

Antibodies used

Cartilage autoantibody cocktail containing 4 arthritogenic monoclonal antibodies (ACC1: anti-citrullinated C1 epitope of collagen type II (COL2) antibody; M2139: COL2 antibody; L10D9: collagen type XI antibody; 15A: anti-cartilage oligomeric matrix protein antibody) (#10040, Vacara)
InVivoMAb anti-mouse IFNAR-1 (MAR1-5A3, BioXCell)
InVivoMAb mouse IgG1 isotype control (MOPC-21, BioXCell)
NF200 (ab4680, Abcam)
CGRP (gift from Tomas Hökfelt)
GFP (ab13970, Abcam)
TH (P40101, Pel-Freez)
IB4 from Griffonia simplicifolia I (GSA I) (Vector Laboratories)
goat anti-GSA I antiserum (Vector Laboratories)
Phospho-P38 (Thr180/Tyr182, # 9211, CellSignaling technology)
Iba-1 (#016-26721, WAKO Chemical)
Phospho-eIF4E (Ser209, #ab76265, Abcam)
Phospho-eIF4E (Ser209, #9741, CellSignaling technology)
eIF4E (#9742, CellSignaling technology)
GAPDH (#5174, CellSignaling technology)
IFN alpha (PA5-86767, ThermoFisher)
beta-Actin (ab6276, Abcam)

Validation

Cartilage autoantibody cocktail (PMID: 32448385, PMID: 37741824)
IFNAR-1 mAb (https://bioxcell.com/invivomab-anti-mouse-ifnar-1-be0241#tab_specifications)
mouse IgG1 isotype control (https://bioxcell.com/invivomab-mouse-igg1-isotype-control-unknown-specificity-be0083)
NF200 (PMID: 30917305), Phospho-P38 (PMID: 16399694), Iba-1 (PMID: 39581686), CGRP (PMID: 25631752), GFP (PMID: 31937758), TH (PMID: 30917305), goat anti-GSA I antiserum (PMID: 25631752), Phospho-eIF4E (PMID: 32245829), Phospho-eIF4E (PMID: 28674170), eIF4E (PMID: 28674170), GAPDH (PMID: 37133968), IFN alpha (PMID: 38303713), beta-Actin (PMID: 12423254)

Animals and other research organisms

Policy information about studies involving animals; ARRIVE guidelines recommended for reporting animal research, and Sex and Gender in Research

Laboratory animals

C57BL/6N mice were ordered from Charles River (Scanbur AB);
Wnt1-cre mice were ordered from The Jackson Laboratory (JAX #003829)
Slc17a8cre was ordered from The Jackson Laboratory (Vglut3Cre, JAX #028534)
Gfra3cre/ERT2 was ordered from The Jackson Laboratory (JAX #029498)
Ifnar1 fl/fl was ordered from The Jackson Laboratory (JAX #028256)
ROSA26Tomato was ordered from The Jackson Laboratory (Ai14, JAX #007914)
ROSA26ChR2-EYFP was ordered from The Jackson Laboratory (ROSA26ChR2, Ai32, JAX #012569)
ROSA26ArchT-EGFP was ordered from The Jackson Laboratory(ROSA26ArchT, Ai40D, JAX #021188)
ROSA26-CAS9 was ordered from The Jackson Laboratory (JAX#028239)
Sstcre was a generous gift from Jens Hjerling-Leffler (JAX #013044)
Mrgprdcre was ordered from Mutant Mouse Resource & Research Centers (MMRRC_036118)
Ntrk1cre/ERT2 (TrkACreERT2) mice were generated in the lab

Wild animals

No wild animals were used in the study

Reporting on sex

Both males and females were used in the behavioural study. However, only males were used for sc-RNA seq data presented in the study

Field-collected samples

No field collected samples were used in the study.

Ethics oversight

All experiments were carried out in accordance with protocols approved by the Stockholm Ethical Committee for Animal Experiments (Stockholms Norra Djurförsöksetiska Nämnd, Sweden)

Note that full information on the approval of the study protocol must also be provided in the manuscript.

Plants

Seed stocks	n/a
Novel plant genotypes	n/a
Authentication	n/a
